# Spent substrate from mushroom cultivation: exploitation potential toward various applications and value-added products

**DOI:** 10.1080/21655979.2023.2252138

**Published:** 2023-09-05

**Authors:** Carlos Martín, Georgios I. Zervakis, Shaojun Xiong, Georgios Koutrotsios, Knut Olav Strætkvern

**Affiliations:** a Department of Biotechnology, Inland Norway University of Applied Sciences, Hamar, Norway; b Department of Chemistry, Umeå University, Umeå, Sweden; c Department of Crop Science, Agricultural University of Athens, Athens, Greece; d Department of Forest Biomaterials and Technology, Swedish University of Agricultural Sciences, Umeå, Sweden

**Keywords:** Spent mushroom substrate, food, feed, biofertilizers, soil amendment, plant-disease control, bioactive compounds

## Abstract

Spent mushroom substrate (SMS) is the residual biomass generated after harvesting the fruitbodies of edible/medicinal fungi. Disposal of SMS, the main by-product of the mushroom cultivation process, often leads to serious environmental problems and is financially demanding. Efficient recycling and valorization of SMS are crucial for the sustainable development of the mushroom industry in the frame of the circular economy principles. The physical properties and chemical composition of SMS are a solid fundament for developing several applications, and recent literature shows an increasing research interest in exploiting that inherent potential. This review provides a thorough outlook on SMS exploitation possibilities and discusses critically recent findings related to specific applications in plant and mushroom cultivation, animal husbandry, and recovery of enzymes and bioactive compounds.

## HIGHLIGHTS

- Valorization of spent substrate is crucial for a sustainable mushroom industry.
- The review covers spent mushroom substrate (SMS) valorization for multiple uses.
- SMS composition and mushroom species are essential factors for its exploitation.
- SMS valorization forms an integral part of cascade use of plant biomass.

## GRAPHICAL ABSTRACT

**Figure:**
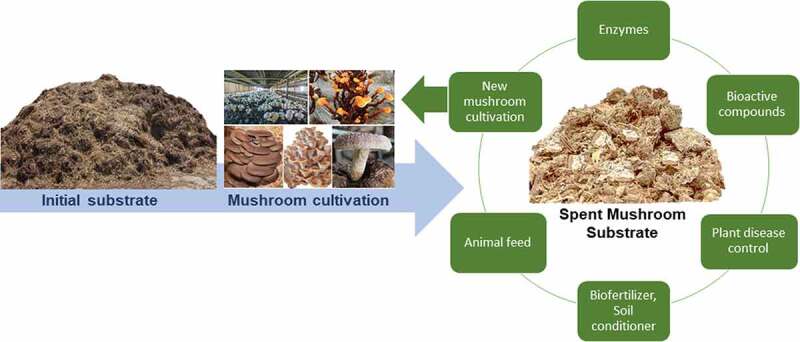

## 1. Introduction

Mushrooms are sporocarps, i.e. visible spore-bearing structures, fulfilling an essential function in the sexual reproductive stage of the life cycle of many fungi [1]. Many mushrooms are considered edible because they do not contain toxins and are low in antinutrients, while they are rich in proteins, dietary fiber, vitamins, minerals, and other nutritional components [2]. The specific composition of mushrooms depends on the species. Mushrooms can have up to 30% (w/w) crude protein, while the content of crude fiber, fat, and carbohydrates in some species can be up to 28, 8, and 95% (w/w), respectively [3]. Edible mushrooms are climate-smart, protein-rich food sources that can partially substitute meat, whose production has a significant climate impact. Furthermore, due to their high content in various health-promoting ingredients, e.g. β-glucans, peptides, proteins, and phenolic compounds [4], they possess immunomodulatory, antibacterial, cytostatic, antioxidant, and other properties, and for this reason, the term ‘medicinal mushrooms’ is also used when referring to them [3].

The benefits of mushroom consumption on human health and wellbeing are well recognized. As a result, pertinent demand has considerably increased in all continents, and edible mushroom commercialization has nowadays become a worldwide business [5]. Hence, mushroom production has increased more than 30 times since 1978, and it is a fast-expanding industrial activity. Although most of the production is concentrated in Asia, with China as the top producer with around 90% of the global market, mushroom production in the European Union, led by the Netherlands and Poland, and in the Americas has experiencing a significant increase during the last decades [6]. The commercial cultivation of mushrooms includes more than fifty species. The top four belong to the genera *Lentinula* (*L. edodes*, popularly known as ‘shiitake’), *Pleurotus* (‘oyster mushrooms’), *Auricularia* (‘wood ear mushrooms’), and *Agaricus* (‘button mushrooms’), which together correspond to 74% of the world market [7].

Mushrooms are cultivated on substrates based on plant biomass, e.g. crop residues and underutilized wood leftovers, which are continuously increasing because of the expansion of agricultural production driven by global population growth. Currently, disposal by burning is one of the chief methods for coping with the accumulation of plant residues. However, this widespread practice is against sustainability principles, contributes substantially to air pollution [8], and results in a considerable waste of biomass resources that are highly valuable for generating materials, fuels, and chemicals of high economic and social value [9].

The valorization of crop residues within new recycling models, i.e. substrates for mushroom cultivation, is crucial for the sustainability of agricultural production. Therefore, besides leading to the generation of food, mushroom cultivation is an example of holistic exploitation of residual lignocellulosic biomass through an efficient continuous-flow process carried out indoors, requiring remarkably lower land areas than most other crops [10]. Furthermore, unlike conventional agriculture, which is season-dependent, mushroom production can be performed throughout the year independently of climatic conditions.

The mushroom cultivation process aims at producing fruitbodies of edible or/and medicinal fungi. At the end of the process, the fruitbodies are harvested, and an exhausted residual substrate is generated. That nutrient-depleted biomass waste, known as spent mushroom substrate (SMS), is the main by-product of the mushroom industry. Depending on the nature of the materials used for formulating the substrate, the type of production system, and the cultivated species, three to five kg of SMS is generated per kg of fresh mushrooms [11]. In total, ca. 64 million tons of SMS were generated worldwide by the mushroom industry in 2018, and this figure could escalate to above 100 million tons by 2026 [12].

The large quantities of generated SMS, currently regarded as a waste product with little inherent value, present a major challenge to mushroom producers due to the need to find suitable disposal sites and to cope with the high cost incurred for the transportation of a bulky material with high moisture content and low density; drying of fresh SMS is a hardly feasible energy-intensive activity. Moreover, SMS handling/disposal is of primary environmental concern due to the emission of greenhouse gases from spontaneous anaerobic digestion (often occurring in the piles formed during provisional storage), foul odors, and leachate drainage to water receptors causing pollution and eutrophication [13]. Landfilling has traditionally been the chief disposal strategy for SMS, but it is now banned in the European Union by a Council Directive on landfilling of biodegradable wastes [14]. The current linear ‘take, make, dispose of’ approach, where SMS is regarded as waste, threatens the future development of the mushroom-growing sector. Valorization of SMS is crucial for developing a sustainable mushroom industry in the frame of a circular-economy model. It is essential to investigate SMS characteristics to identify appropriate valorization alternatives.

SMS composition and properties are mainly associated with the type of raw materials and supplements used to prepare the initial mushroom substrate. For the cultivation of edible mushrooms of the genera *Lentinula*, *Pleurotus*, and *Auricularia*, which represent 60% of the global production, various lignocellulosic by-products, e.g. forest, agricultural and agro-industrial residues, are used as substrate base. Chicken manure is also a major component for other mushroom species requiring composted substrates (e.g. those of the genus *Agaricus*). Starch-containing and nitrogen-rich ingredients (e.g. cereal bran or legumes’ flour) and mineral salts are used as supplements. During cultivation, substrate components are enzymatically degraded, and the resulting nutrients (together with others existing in the substrate) are used for fungal growth and mushroom production. Mass losses in the ranges of 26–46%, 57–77%, and 61–75% of the initial cellulose, hemicelluloses, and lignin, respectively, have been reported for *Pleurotus ostreatus*, *Pleurotus pulmonarius*, and *L. edodes* [15–17]. In the end, SMS composition strongly depends on the nature of the initial substrate and the cultivated species [18]. Therefore, SMS primarily consists of plant cell-wall components (lignin, hemicelluloses, cellulose) and residual fungal mycelium, as well as non-cell-wall carbohydrates, proteins, and minerals.

There are different valorization routes for SMS, and some of them have already been discussed in previously published reviews [19,20]. The current review is aimed at providing, in brief, an updated overview of potential SMS applications and products related to (i) new cycles of mushroom cultivation, (ii) agriculture and animal husbandry, and (iii) the production of enzymes and bioactive compounds. SMS valorization as part of cascade-use systems for plant biomass processing is also discussed. Bioremediation and energy-related uses are not included because they were exhaustively presented in a recent review [20]. This review is based on an exhaustive Scopus search performed in July 2022. The search terms used were Spent Mushroom Substrate OR Spent Mushroom Compost AND relevant keywords of each specific application. The topic presented in this review is of relevance to the UN Sustainable Development Goals 2 (Zero hunger), 3 (Good health and well-being), 9 (industry, innovation, and infrastructure), 13 (climate action), and 15 (life on land), considering that the discussed valorization alternatives have the potential for providing innovative solutions to increase food security, and contributing to the production of healthy food and reduction of the use of harmful chemicals in farmlands.

## 2. Reusing spent mushroom substrate for new cultivation of mushrooms

The spent mushroom substrate can be used in substrate formulation for new cycles of mushroom cultivation provided that suitable lignocellulosic materials are employed, the fungal strain is appropriately selected, and the environmental conditions are optimally regulated. Supplemented cereal straw and wood sawdust are the most common substrates in commercial mushroom cultivation due to their composition, availability, and relatively low cost. Agricultural or agro-industrial by-products with low or no economic value, such as sugarcane bagasse, coffee husks, and olive mill and winery wastes, are exploited in mushroom production, contributing to both the improvement of cultivation performance and the enhancement of mushrooms nutritional value [21–24]. Using cheap lignocellulosic residues positively affects the cost of substrate, providing an environmentally friendly solution for their effective management and valorization.

**Figure 1.**
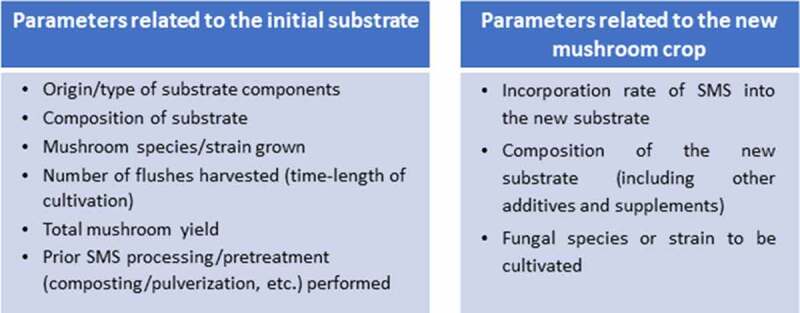
Factors affecting cultivation parameters and the use of SMS in new mushroom crops.

Cultivated mushrooms are often grouped – based on their ecological adaptation/requirements – as either primary decomposers (e.g. *P. ostreatus, L. edodes*) that are produced directly on previously untreated (or partly treated/composted) lignocellulosic substrates, or as secondary decomposers (e.g. *Agaricus bisporus, Volvariella volvacea*). Secondary decomposers are cultivated on composted substrates prepared from various agricultural residues, including manures. The proposal to reuse SMS in new crops was originally based on the sequential use of the substrate, first by primary decomposers and then by secondary decomposers, and on the enzymes involved in each process since these vary among species of different ecological groups [20,25]. However, in most studies, supplementation is required to adjust the nutrient content when SMS is used as the sole (or the main) substrate ingredient in mushroom cultivation. Hence, this material could be exploited to cultivate a broader range of mushroom species (not only secondary decomposers). Furthermore, many of the most successful SMS applications have been reported when the same species (as the one originally cultivated on the spent substrate) was also used in the new crop, e.g. *P. ostreatus, Auricularia polytricha, A. bisporus* [26–29]. The initial substrate composition, the cultivation cycle duration, and the number of flushes harvested are important in optimizing SMS for reuse in mushroom cultivation. The type of substrate pretreatment adopted (e.g. chopping, composting) prior to cultivation, the incorporation rate of SMS to the main substrate of the new crop, further supplementation with nutrients, and the selection of the species/strain to be used are also important parameters, which have to be considered when such types of applications are developed. Factors affecting the success of new mushroom crops based on SMS recycling are summarized in Figure 1.

Reported results from using SMS in new mushroom crops demonstrate a wide variation as regards the effect of the recycled material on the final yield (Table 1). In several cases, similar [27,29,40,44,50] or even higher [26,28,45] values of biological efficiencies (ΒΕ; percentage ratio of fresh mushroom weight over the dry weight of the respective substrate) were recorded in substrates containing SMS deriving from the cultivation of either the same or other mushroom species when compared to conventional (used for the first time) substrates. However, in some studies, it was also reported that the incorporation of high amounts of SMS in the new cultivation medium or the casing layer negatively affected the mushrooms’ final yield [30,36,37,42], which could be mainly attributed to the low content of nutrients or to inadequate supplementation of the spent substrates.

**Table 1.** Reuse of spent mushroom substrate (SMS) for the cultivation of various mushroom species as reported in pertinent publications: origin and composition of SMS, mushroom to be cultivated, new substrate formulation and supplements, biological efficiency (BE) reported for the crop obtained and main comments on the results of the respective study. Abbreviation used: NR, not reported.

Origin of SMS	Composition of SMS	Mushroom to be cultivated	New substrate formulation	Supplements to new substrate	Biological efficiency	Comments	References
*Pleurotus ostreatus*	NR	*P. ostreatus*	SMS (plain, 3:1, 1:1 and 1:3) with rubber sawdust	CaCO_3_ (1.5% w/w), wheat bran (5% w/w)	53–56%	Similar BE values with the control (rubber sawdust; BE: 53%)	[29]
*Pholiota nameko*, *Hypsizigus marmoreus*, *Hericium erinaceus*	Oak and beech sawdust (1:1), wheat bran (20%), corn flour (5%) and gypsum (1%)		SMS and wheat straw (1:9, 2:8 and 3:7)	Wheat bran (3%), gypsum (1%)	66–73%	Reduction in BE was noted when SMS ratios increased	[30]
		*P. ostreatus*					
*P. ostreatus*	Wheat straw, wheat bran, soybean flour and CaCO3	*P. ostreatus*	SMS	Wheat bran, soybean flour, and CaCO_3_ (C/N: 20)	185%	High (the highest reported) BE values for both species examined by using SMS as cultivation substrate	[26]
		*Pleurotus pulmonarius*			208%		
*Cyclocybe cylindracea*, *Auricularia polytricha*, *H. erinaceus*, *Lyophyllum shimeji*, *Pleurotus citrinopileatus*, *Pleurotus cystidiosus*, *Pleurotus eryngii*, *P. ostreatus*, *Pleurotus sajor-caju*	Sawdust	*A. polytricha*	SMS from various species	Rice bran (9.5%), CaCO_3_ (.5%)	41–59%	All mixtures exhibited higher BE values than the control (BE: 36%)	[28]
*Flammulina velutipes*	NR	*Cordyceps militaris*	SMS:wheat bran: rice bran (8:1:1)	Glucose (20 g L^−1^) and peptone (5 g L^−1^)	35%	Lower yields and cordycepin concentration in SMS-based substrate than in conventional substrate	[31]
*P. eryngii*	Sugarcane bagasse (50%), cottonseed hulls (20%), wheat bran (20%), cornmeal (5%), soymeal (3%), lime (1%), gypsum (1%)	*C. cylindracea*	SMS	Sucrose (1%), lime (1%), wheat bran (0, 10, 20%), and *Tenebrio molitor* feces (0, 10, 20%)	40–63%	Increasing the rate of supplements positively affected the ΒΕ	[32]
*P. pulmonarius*	Rubber sawdust supplemented with 20% palm oil sludge	*C. cylindracea*	SMS alone or mixed with rubber sawdust (3:1, 1:1 and 1:3)	Rice bran (5%), Ca(OH)_2_ (2%), Mg(SO_4_) (.2%)	25–41%	BE increased by increasing SMS content	[33]
*Agaricus bisporus*	NR	*P. ostreatus*	*P. ostreatus* SMS, and *A. bisporus* SMS (various ratios)	CaSO_4_ (5%), gypsum (5%)	NR	*P. ostreatus* and *A. bisporus* SMS (3:2 and 1:1) had similar protein and lower ash content than commercial substrate; SMS affected the produced mushrooms’ quality	[34]
*P. ostreatus*	NR						
*P. ostreatus*	NR	*P. ostreatus*	SMS or SMS:wheat straw (1:1)	Wheat bran (50 or 100 g kg^−1^), Calprozime (20 g kg^−1^), gypsum (5%), CaCO_3_	3–62%	Reduction in BE by increasing wheat bran supplementation; highest BE values were obtained using Calprozime	[35]
*H. marmoreus*	NR	*P. ostreatus*	Cottonseed hulls:SMS:wheat bran (various ratios)	Wheat bran (0–18%), lime (1%), gypsum (1%)	36–61%	Reduction in BE when SMS content was increased	[36]
*P. ostreatus*	NR	*P. ostreatus*	SMS:sawdust (7:3, 6:4, 5:5, 4:6 and 2:8)	Wheat bran (20%)	78–105%	Reduction in BE when SMS ratio is increased	[37]
		*Pleurotus florida*			67–89%		
NR		*P. eryngii*	SMS:farmyard manure (1:1) and SMS as casing material (3 cm thick)		49–107%	Enhanced yield on SMS compared to other casing materials and non-casing substrates	[38]
*P. eryngii*	Sawdust (50%), cottonseed hulls (25%), wheat bran (25%), gypsum and CaCO3	*Volvariella volvacea*	Composted or non-composted SMS	Limestone (5%)	10–17%	Composted SMS performed better (higher BE) than non-composted one	[39]
*F. velutipes*	NR	*A. bisporus*	SMS	Gypsum (2.83%), CaH_4_P_2_O_8_ (2.83%)	29%	Similar BE values with the control (rice straw), but SMS results in shorter production cycles	[40]
*P. ostreatus*	NR	*Agaricus blazei*	Sunflower seed hulls:SMS (milled or unmilled)		13.1 – 22.4%	Milled SMS reduced BE	[41]
*A. bisporus*	Wheat straw and poultry manure-based commercial compost	*A. bisporus* casing	Sphagnum peat:SMS (4:1, 3:2. 2:3, 1:4) and plain SMS	CaCO_3_ (100 g L^−1^)	53–110%	Lower BE (than the control) when SMS was incorporated in the casing layer	[42]
*P. ostreatus*	Banana leaf straw	*A. blazei*	SMS with burned rice husk or subsoil as casing material	CaCO_3_, urea, rice bran (1, 10 or 20%), (NH)_2_SO_4_	0–80%	Highest BE values obtained with rice bran (10%) and with subsoil as casing material	[43]
*P. sajor-caju*							
*P. ostreatus* and *A. bisporus*	NR	*P. ostreatus*	*P. ostreatus* and *A. bisporus* SMS (9:1 and 8:2)		36–40%	Comparable BE values with those obtained using a wheat straw and poultry manure-based commercial substrate	[44]
*Pleurotus eous*	Wheat straw	*P. sajor-caju*	SMS with wheat straw (1:9, 1:6 and 1:3)		62–69%	BE increased by increasing SMS content and was higher than the control (wheat straw)	[45]
		*P. florida*			109 – 113%		
		*Pleurotus flabellatus*			97 – 105%		
*A. bisporus*	Commercial compost with casing	*A. bisporus*	SMS	Soybean meal (10%), Target® (10%)	26–73%	Lower BE values compared to the conventional substrate	[27]
			SMS and non-composted substrate (1:1 w/w)		97–144%	Similar or higher BE values in comparison to the conventional substrate	
*Lentinula edodes*	Sawdust	*P. citrinopileatus*	SMS alone and mixed with *Mangifera indica* sawdust (1:2 w/w)	Rice bran (10%), CaCO_3_ (pH 6)	23–39%	Increased BE noted when SMS was mixed with *M. indica* sawdust	[46]
*P. sajor-caju*					19–34%		
*P. sajor-caju*	Rice straw	*P. sajor-caju*	SMS	Mustard, niger, sunflower, cotton seed powder and soybean seed cake	45–125%	Better mushroom yields with cotton seed powder than with the other supplements	[47]
*F. velutipes*	NM	*L. edodes*	SMS and SMS:oak sawdust (1:4, 2:3, 3:2, 4:1)	Rice bran (20%)	60–84%	High BE values for sawdust ratios above 40%	[48]
*A. bisporus*	NR	*L. edodes*	SMS:oak:spruce (1:1:1) and plain SMS	Wheat bran (3%), millet (35%) and rye (2%)	33–53%	Higher BE values for the mixture than for plain SMS	[49]
*L. edodes*	Sawdust	*Pleurotus cornucopiae*	SMS:rice bran (1:1)		61%	BE values higher than the control (*Abies sachalinensis* sawdust) only for *P. cornucopiae*	[50]
		*P. ostreatus*	SMS:rice bran (1:1)		62%		
		*F. velutipes*	SMS:wheat bran (1:1)		88%		
*P. ostreatus*	*Cryptomeria japonica* sawdust	*Lyophyllum decastes*	SMS and SMS:bark compost (4:1 and 1:1)	Rice bran 10%	62–134%	BE two-fold higher for mixtures plain SMS	[51]
*Ph. nameko*	Harwood sawdust				140–167%	No significant differences in BE	
*L. edodes*	Oak sawdust	*P. sajor-caju*	SMS	Soybean flour (0 or 12%), CaCO_3_ (0 or 1%)	62–79%	Higher BE values with soybean flour supplementation	[52]

Most of the investigations related to the reuse of SMS in new cultivation cycles focus on species of the genus *Pleurotus*. Indicatively, out of the 27 selected studies shown in Table 1, 16 deal with the reuse of *Pleurotus* SMS, and 14 with the cultivation of oyster mushrooms in SMS-containing substrates. This may be explained by the relative ease of oyster mushroom cultivation, their rather short production cycle, and the wide range of suitable substrates available. Among the most relevant examples are those pertaining to the use of *Pleurotus eous* SMS mixed with wheat straw for the cultivation of other *Pleurotus* species (BE up to 113% [45]) and the use of supplemented SMS from *Pleurotus sajor-caju* for the production of the same mushroom (BE up to 125% [47]). Another successful example is the use of supplemented SMS from *P. ostreatus* as substrate for growing *P. ostreatus* and *P. pulmonarius* mushrooms, which resulted in the highest BE values reported in pertinent literature, namely 185% for *P. ostreatus* and 208% for *P. pulmonarius* [26]. Furthermore, *Pleurotus* SMS can also be exploited in crops of other species providing satisfactory yields, as it is the case in *Lyophyllum decastes*, where the use of *P. ostreatus* SMS with bark compost and rice straw provided BE of 134% [51]. In contrast, cultivation of *Pleurotus* species on spent substrates from other mushrooms, i.e. *Pholiota nameko*, *Hypsizigus marmoreus*, and *L. edodes* exhibited substantially lower BE (23–62%) [36,46,50].

Shiitake (*L. edodes*), a widely cultivated edible mushroom, is produced mainly on hardwood sawdust substrates. SMS deriving from *L. edodes* seems to be suitable for the cultivation of various oyster mushroom species, including *P. ostreatus*, *P. sajor-caju* and *Pleurotus cornucopiae* (BE: 61–79%) as well as for *Flammulina velutipes* (BE 88%) following rich supplementation with cereal derivatives [50,52]. On the other hand, using SMS from other mushrooms (e.g. *A. bisporus* and *F. velutipes*) to cultivate shiitake requires mixing with untreated sawdust at a rate of at least 40% [48,49].

The reuse of SMS deriving from the cultivation of *Agaricus* species to establish new mushroom crops is quite demanding due to the nature of the final material and the processes leading to its production. Therefore, most attempts focus either on suitably upgrading it with soybean meal and Target® (a commercial delayed release nutrient suitable for mushroom cultivation) before reusing it as an ingredient for *A. bisporus* cultivation (ΒΕ: 97–144%) [27]. Attempts focusing on exploiting SMS as casing material alone or mixed with farm yard manure or sphagnum peat in the cultivation of *Pleurotus eryngii* and *A. bisporus*, respectively, have also been reported [38,42].

Concerning the use of SMS of less widespread species, noteworthy is the case of *F. velutipes* SMS – which when combined with oak sawdust and rice bran – exhibited satisfactory yields of *L. edodes* mushrooms (BE: 60–84%) [48]. In addition, the sawdust-based SMS from *Ph. nameko*, *H. marmoreus* and *Hericium erinaceus* were used to produce *P. ostreatus* mushrooms (BE: 66–73%) [30].

The reuse of SMS in new mushroom crops seems to have considerable potential since it can support high yields and is both financially feasible and environmentally sustainable. The elements and organic compounds existing in SMS constitute valuable sources of energy and nutrients, which can partially or entirely cover the needs of additional cultivation cycle(s) after suitable treatment or supplementation.

## 3. Spent mushroom substrate as feed

It is estimated that agricultural production should be increased by 70–100% to meet the food demand of the increasing global population, which is predicted to grow to 9.7 billion by 2050 [53]. Soybeans and maize are the most common energy and protein sources used by livestock farmers to generate meat, eggs, and dairy products, which, in turn, are the main protein sources in human diet [54]. The need for animal feed production is predicted to increase significantly, and the feed industry must look for additional/alternative means to cover the respective demand. Exploiting suitable bioresources (e.g. SMS) could contribute toward this direction by readily providing material to be used as feed supplement.

The main raw materials used in mushroom cultivation are rich in cellulose, hemicelluloses and lignin, while their protein content is generally low [23]. During solid-state fermentation by mushroom-forming fungi, the substrate polymers are enzymatically degraded, and the digestibility of plant residues is considerably improved. Concomitantly, the growth of mycelial biomass upgrades the substrate by increasing its content in proteins and bioactive compounds, e.g. polysaccharides and ergosterol [55–58]. Indicatively, the growth of *P. ostreatus* mushrooms on faba bean hulls increased their protein content from 208 g kg^−1^ (on a dry weight (DW) basis) in the initial substrate to 347 g kg^−1^ in the SMS [59]. Furthermore, *P. ostreatus* growth enriched the material in 14 out of 16 analyzed amino acids, and significantly reduced the content of anti-nutritional compounds, such as tannin, vicine, and convicine. SMS from other mushroom species is also rich in compounds of interest for enhancing the quality of feed rations. *L. edodes* SMS is rich in the provitamin D_2_ ergosterol (151.6 mg ergosterol equivalents/100 g) [58], while SMS from several other species contains high amounts of polysaccharides, including β-glucans [60]. Consequently, the high nutritional value of SMS is the main factor for its inclusion in the diets of poultry, ruminants, and monogastric animals, and, recently, in fish and edible insects. A summary of relevant reports on the reuse of SMS as animal feed is presented in Table 2. In addition, the main outcome of each study is briefly presented and further discussed below.

**Table 2.** Reuse of spent mushroom substrate (SMS) as animal feed based on the outcome reported in pertinent publications. Abbreviations used: ADF, acid detergent fiber; ADL, acid detergent lignin; HWE, hot water extract; NDF, neutral detergent fiber; NR, not reported; S-SMS: steam-treated SMS; UN-SMS: untreated SMS.

Origin of SMS	SMS content in fibers and crude protein			Animals in feeding trials	Type of feed, inclusion levels, and length of feeding trials	Main outcome	Ref.
	NFD, ADF and ADL (%)	Crude fiber (%)	Protein (%)				
*Pleurotus sajor-caju*	60.3, 52.3, 4.1	NR	9.3	Alpine dairy goats	Rice straw fermented with *P. sajor-caju* SMS (5:1 w/w for 8 weeks), diet and water provided *ad libitum*, 28 days	SMS effectively improved the nutrient content, availability in the rumen, and feeding value of rice straw; increased effective degradability of dry mater and fibers in the rumen; improved intake and milk yield	[61]
*Pleurotus ostreatus*	NR	NR	3.0	Male Sika deers	SMS (10%), 60 days	SMS led to reduction in the intake of organic matter, and improved digestibility of crude fat	[62]
*Flammulina velutipes*	NR	NR	5.1			Νo effect on apparent nutrient digestibility, feed intake, velvet antler production, or biochemical indexes	
*P. ostreatus*	NR	NR	NR	Liuyang black goats	SMS co-fermented with feed and whole plant rice, 60 days	Feeding with co-fermented whole plant rice and SMS had no adverse effect on the slaughter performance, while the meat quality was improved	[63]
*Cordyceps militaris*	NR	NR	NR	Crossbred growing pigs	SMS (0.2%, w/w), 6 weeks	SMS increased final body weight and daily weight gain, immunoglobulin A and G, and the rest of the parameters were not affected.	[55]
*Lentinula edodes*	NR	NR	NR	Weaned piglets	SMS (3%) fermented by *Bacillus subtilis*, 33 days	Increase in final weight, daily gain and feed conversion; beneficial effect on the intestinal mucosal barrier, and immunity	[64]
*Ganoderma lucidum*	NR	NR	23.6	Mice	HWE of SMS (0.14, 0.28, 0.84 and 1.68 g kg^−1^), 30 days	HWE of SMS enhanced murine immune function; the 0.84 g/kg dose had optimal effect in all aspects	[65]
*P. ostreatus*	74.8, 49.4, NR	24.2	7.9	Hanwoo steers	SMS fermented or not with *Lactobacillus brevis*, 13 days	SMS (fermented or not) could replace formulated feed concentrate without adverse effects	[66]
*P. ostreatus*	73.6, 55.0, NR	NR	8.1	Geese	SMS fed *ad libitum* during the growing period, 8 weeks)	SMS supplementation at 5% had no adverse effects on the growth performance, while it favorably affected sensory attributes	[67]
*Pleurotus eryngii*	50.6*, 38.9*, 11.7*	31.2*	16.9*	Sheep	By-product feed silage with fermented SMS (45%), 22 days	The SMS-containing feed showed similar energy value, higher protein metabolism and utilization, and lower fiber digestion than the rye straw-based control diet	[68]
*P. ostreatus*	NR	NR	5.6	Awassi sheep	SMS (5, 10, 15 and 20%), 70 days	SMS ratios (>15%) decreased slaughter, empty body, and carcass weights, dressing, leg lean and fat tail percentage, backfat thickness, and rib eye area	[69]
*P. ostreatus*	76.7, 61.2, 15.5	NR	13.4	Hanwoo steers	Feed containing 50% SMS; *ad libitum* access during the growing and fattening periods	Trials with by-product feed tended to increase the average daily weight gain and feed efficiency, while it did not affect quality and yield traits	[70]
*G. lucidum*	NR	6	23.6	Holstein cows	HWE of SMS (33, 67, and 100 g d^−1^ cow^−1^), 60 days	HWE of SMS may enhance immunity and antioxidant capacity in dairy cows, and subsequently improve milk quality	[71]
*Ganoderma chalceum*(syn. *G. balabacense*)	NR	NR	23.6	Chinese Holstein cows	HWE of SMS (33, 67, and 100 g d^−1^ cow^−1^), 60 days	Feeding by HWE of SMS improved hematology parameters, and increased milk yield, milk protein and triglyceride levels	[72]
*Agaricus bisporus*	27.8, NR, 6.9	13.3	12.9	Holsteins male calves	SMS (15%), 170 days	No differences detected in the carcass and internal organs of the calves that received different diets	[73]
*P. ostreatus*	NR	29.6	7.9	Broilers	SMS substituted wheat bran (by 25–100%), 8 weeks	Higher feed intake with increased rate of SMS inclusion; SMS did not affect breast, thigh drumstick, back, neck, wings, and shoulder weight	[74]
*Hypsizygus marmoreus*	NR	-	-	Laying hens	SMS (5, 10 and 15%) fermented with *Bacillus subtilis*, 12 weeks	Feed intake increased with SMS addition; no differences in egg production, egg weight, egg mass, feed conversion and viability; the yolk color was more intense when SMS was added	[75]
*Grifola frondosa*	Steam-treated SMS (S-SMS) and non-treated SMS (N-SMS)	NR	6.6(S-SMS), 6.4 (N-SMS)	Wistar rats	S-SMS or N-SMS (25%), 26 days	Feed with SMS did not affect body weight gain, feed efficiency, or serum biochemical parameters; however, fecal weight and protein content were significantly higher than the control	[76]
*P. eryngii*	78.8, 66.0. 12.8	NR	5.7	Hanwoo steers during growing and fattening periods	*Ad libitum* access to microbially-fermented SMS (50%), 12.6 months	Feeding with microbially fermented SMS improved growth performance and carcass traits, and could successfully replace a part of conventional roughage	[77]
*P. ostreatus*	65.1, 49.4, NR	40.5	15.4	Postweaning calves	SMS (10%) fermented or not with lactic acid bacteria, 60 days	The fermented SMS improved the growth performance compared to non-fermented SMS and to feed with an antibiotic supplement	[78]
*P. eryngii*	NR	NR	NR	Laying hens	SMS (5, 10 and 15%) fermented with *Bacillus subtilis*, 7 weeks	Feed intake increased with SMS addition; no differences noted in egg production, egg weight, egg mass, feed conversion and viability; yolk color was more intense when SMS was added	[79]
*P. sajor-caju*	NR	NR	6.3	Broiler chicken	SMS (0.5 to 2%), 21 or 38 days	The inclusion of SMS up to 0.67% improved the weight gain of broiler chicks in the first 21 days	[80]
*Agaricus blazei*	NR	NR	NR	Broiler chicks	SMS (0.2 to 1.0%), 42 days	SMS above 0.4% reduced the animals’ performance; 0.2% SMS resulted in the highest weight gain and feed intake, and the best feed conversion	[81]
*P. ostreatus*	NR	9.4	17.6	Berkshire pigs	SMS (plus rice bran and barley bran, 2:1:1) in ratios of 3, 5 or 7%, 7 weeks	Daily feed intake and feed conversion increased through SMS addition; SMS (3%) positively affected the growth performance, carcass traits, meat quality and fatty acids concentration in meat	[82]
*A. bisporus*	27.8, 21.0, 6.8	17.8	11.0	Sheep	SMS (10, 20, 30%), 3 weeks	Up to 20% SMS did not affect nutrient intake, digestibility, or nitrogen balance	[83]

*Values referrer to bioproduct-feed with SMS (45%).

### 3.1. Spent mushroom substrate in the diet of poultry

The incorporation of SMS derived from the cultivation of *P. eryngii*, *P. ostreatus* and *H. marmoreus* (fermented or not by *Bacillus subtilis*) at a ratio from 5 to 15% (w/w) in a poultry diet increased the feed intake without having adverse effects on the egg production and the mass of useful meat [67,74,75,79]. On the other hand, incorporation of *Agaricus blazei* SMS at rates exceeding 0.4% (w/w) caused a gradual reduction in the weight gain of broiler chickens, while inclusion ratios of only 0.2% exhibited the highest value of weight gain and feed intake, as well as the best feed conversion [81]. Similarly, low inclusion ratios of *P. sajor-caju* SMS (up to 0.67%) improved the weight gain of broiler chicks in the first 21 days [80].

### 3.2. Spent mushroom substrate in the diet of monogastric animals

SMS inclusion in the diet of monogastric animals have been tested with both pigs and model animals (mice, rats). The addition of sawdust-based SMS from *Grifola frondosa* (25% w/w) in rats’ diet did not affect the weight gain, the feed efficiency, or the biochemical parameters, while fecal weight and protein content were found to be higher [76]. In addition, the – orally administered – hot water extract of SMS from *Ganoderma lucidum* exhibited enhanced murine immune function in mice [65]. Furthermore, the use of low amounts of SMS from *P. ostreatus* (up to 3.5%, w/w), *Cordyceps militaris* (0.2%, w/w), and *L. edodes* (3%, w/w) in pigs’ diet positively affected the feed intake and conversion, as well as the final weight and quality of meat deriving from trials [55,64,82]. *C. militaris* SMS resulted in increased immunoglobulin A and G, and glutathione peroxidase activities, while leukocytes, cholesterol and malondialdehyde contents were decreased [55]. Similarly, beneficial effects on the intestinal mucosal barrier, immunity, and the diversity and abundance of the bacteria in the colon and cecum were observed for weaned piglets when fed with *L. edodes* SMS [64]. It is noteworthy that SMC seems to be useful as a ‘behavior regulator’ in pigs; by having access to mushroom compost through a metal grid, pigs demonstrated significantly reduced negative behavior (e.g. such as nosing, tail biting and chewing) against penmates, as well as improved overall welfare in comparison to pigs with no access to SMC [84].

### 3.3. Spent mushroom substrate in the diet of ruminants

The use of SMS as animal feed has been investigated to a larger extent for ruminants than for monogastric animals (i.e. 13 vs. 5 publications appeared, respectively, when a Scopus search was performed by using the keywords ‘Spent Mushroom substrate/compost’ AND ‘feed;’ July 2022). Incorporating SMS from various mushroom species at a rate of up to 30% (w/w) in the daily intake of ruminants revealed its potential as a supplement to conventional feeds without affecting several relevant parameters (Table 2). Specifically, feeding sheep for three weeks with a diet including up to 20% (w/w) of *A. bisporus* SMS did not affect the nutrient intake, digestibility, and nitrogen balance [83]. Similarly, *A. bisporus* SMS fed at a rate of 15% (w/w) for 170 days did not cause any effect on the carcass and internal organs of Holsteins male calves [73]. Plain *P. ostreatus* SMS in ratios higher than 15% resulted in adverse effects in sheep slaughter weight, empty body weight, and hot and cold carcass weight [69]. In contrast, when rice straw was fermented for eight weeks with *P. sajor-caju* SMS before being fed to alpine dairy goats, it increased the rumen degradable fibers fraction and improved dry matter intake and milk yield [61].

Feeding male sika deer for 60 days with *P. ostreatus* SMS (10%, w/w) resulted in a reduction of the intake of organic matter and improved digestibility of crude fibers, while no effect on either the apparent nutrient digestibility, feed intake, velvet antler production, or biochemical indexes was observed when *F. velutipes* SMS (10%, w/w) was fed to the same animals [62]. When hot water extracts from *G. lucidum* and *Ganoderma chalceum* (syn. *G. balabacense*) SMS were supplemented to dairy cow feed, immunity and antioxidant capacity were increased, and milk quality was improved [71,72]. By using SMS extracts, the addition of large amounts of fibrous components from the untreated SMS could be avoided, but further studies are required to investigate their impact on animal health and the optimum incorporation rate, which depends mainly on the substrate origin.

*Agaricus* and *Pleurotus* species are usually cultivated in straw-based substrates, while *L. edodes*, *G. lucidum*, *Gr. frondosa* and *He. erinaceus* are cultivated in wood-based substrates. The incorporation rate of such substrates in animal feed is low, and further treatment is necessary to improve their nutritional characteristics. In recent years, microbial fermentation with probiotic microorganisms has been adopted as a cheap, fast, and efficient method to reduce fibrous ingredients and upgrade the protein content of SMS, including those deriving from sawdust-based media. Moreover, probiotic microorganisms relieve animals weaning stress, regulate intestinal microbiota, and reduce diarrhea incidents [85,86].

Due to its high moisture content, SMS tends to decompose rapidly; hence, it needs to be processed quickly. This could be achieved by ensiling, for instance, by lactic acid fermentation under anaerobic conditions [87]. Lactic acid bacteria produce desirable metabolites, and suppress the growth of clostridia and other deleterious microbial populations [88]. Although ensiling processes may be initiated naturally by the epiphytic microorganisms existing in the initial material, they can be assisted by inoculated bacteria. Inoculation of SMS with *Lactobacillus*, *Bacillus*, or *Enterobacte*r spp. ensures rapid acidification, and increases dry matter degradability and crude protein content [64,66,70,75,77,78].

An indicative example is the use of a sawdust-based *P. eryngii* SMS incorporated at a high rate (45%, w/w) into silage with various agricultural by-products, and fermented for 22 days [68]. Using the resulting product for feeding sheep resulted in similar energy value, lower digestion of fibers, and higher protein metabolism and utilization compared to the outcome achieved with a rye straw-based control diet. In addition, the sawdust-based SMS from the same mushroom species, when fermented with *Enterobacter* and *Bacillus* spp., significantly improved growth performance and carcass traits in Hanwoo steers compared to rice straw feed administered for 12.6 months during the growing and fattening periods [77]. Similarly, *P. ostreatus* SMS fermented with *Lactobacillus plantarum* and *Pediococcus acidilactici* could replace up to 50% of the conventional feed provided to Hanwoo steers and postweaning calves [66,70,78]. Such an SMS-based feed improved the growth performance of the tested animals or enhanced the daily gain caused by increased voluntary feed intake. Finally, feeding Liuyang black goats with *P. ostreatus* SMS co-fermented with whole rice plants improved meat quality and had no adverse effects on the slaughter performance [63].

### 3.4. Spent mushroom substrate in the diet of fish and edible insects

Using SMS in pisciculture is also of substantial interest. SMS from *P. ostreatus*, *Pleurotus cystidiosus*, and *G. lucidum* seems to support the growth of catfish, and significantly increase its survival rate and digesting ability compared to commercial feeds [89]. The addition of *C. militaris* SMS up to 40 g kg^−1^ in the diet of Nile tilapia (*Oreochromis niloticus*) improved growth performance, skin mucus lysozyme, and peroxidase activities, as well as serum immune parameters [90]. The combination of SMS with *L. plantarum* further improved those parameters. Moreover, enrichment of Nile tilapia diet with *A. blazei* SMC (1%, w/w) provided significant protection against infections from *Streptococcus agalactiae* [91]. Including an extract from the SMS of *Schizophyllum commune*, a popular mushroom in Thailand, in the feed of Nile tilapia resulted at enhancing their immune defense [92].

In the frame of the need to reduce dependence on feeds deriving from plants, the use of insects seems to be a promising alternative due to their high content in crude protein (up to 76%) and fat (up to 59%), energy (20–30 MJ/kg DM), as well as to their short life-cycle and the low-cost growth prerequisites [93–95]. The potential of six SMS derived from *Auricularia cornea*, *Auricularia heimuer*, *P. eryngii*, *P. ostreatus*, *Pleurotus citrinopileatus*, and *L. edodes* was recently evaluated to rear black soldier fly (*Hermetia illucens*) and *Tenebrio molitor* larvae. *L. edodes* SMS was shown to be the most suitable to replace the insects' conventional feed [96,97]. Furthermore, when *Protaetia brevitarsis* larvae were grown on *L. edodes* or *Auricularia auricula-judae* SMS, a nutrient-rich organic fertilizer with low phytotoxicity and high humic acid content was produced [98]. However, no studies exist on the production of insects naturally feeding on mushroom substrates. That could be a promising alternative considering the ease of insects’ growth and the low demand in terms of material resources.

In conclusion, using SMS in animal nutrition can significantly contribute to the enrichment of feed, particularly regarding proteins and bioactive compounds. However, incorporating SMS into the daily feed-schedule is a complex process. The mushroom species, the initial substrate composition, the animal species, and the digestibility and voluntary intake of the final product are factors that must be carefully considered to calculate the final integration rate. The high NDF and ADF content (especially in sawdust-based substrates) is probably the main limiting factor in SMS exploitation as feed. Adopting appropriate treatment approaches, including lactic acid fermentation and the use of SMS extracts, could enhance the nutritional and acceptance characteristics, thus facilitating the incorporation of SMS in the diet of productive animals. Particularities related to the composition of each type of SMS and the individual needs of the animal species require careful experimentation on a case-by-case basis to ascertain the safe and efficient use of SMS.

## 4. Use of spent mushroom substrate in agriculture

The global demand for food and feed has led to intensification of agricultural production and the widespread use of fertilizers and pesticides. World consumption of the three main fertilizer elements (Ν, P, K) was estimated at 201.7 million tons in 2020 [99], and nearly 3 billion kg of pesticides are used yearly [100]. Although using fertilizers and pesticides has increased food availability, their extensive application negatively impacts the environment and human health. Hence, adopting sustainable agronomic practices, including the development of novel environment-friendly and cost-effective biofertilizers and biopesticides, is of high priority. In line with that approach, the SMS’s physical properties, its high content of bioactive compounds, and readily available macro- and trace elements make it a promising candidate for several agricultural applications, the most important of which are presented in the following paragraphs.

### 4.1. Use of spent mushroom substrate as biofertilizer and soil conditioner

Organic soil amendments, commonly used in agriculture, exert positive effects on crop productivity and soil health by affecting physicochemical and biological properties of soil [101–103]. Among the most widespread materials used as organic soil amendments are those originating from municipal wastes (food and gardening wastes, sewage sludge), animal husbandry (manure), crop production (stems, leaves and branches), and agro-industrial activities (fruit pulp and oil extraction by-products). However, those materials can contain hazardous compounds or plant pathogens, which are detrimental to soils and crops.

Since SMS is rich in nutrients and has low (and, most often, no) content in xenobiotic compounds and heavy metals, it can be used as a soil amendment either directly or after a composting treatment. SMS properties vary depending on the raw materials included in the initial substrate, the mushroom species, and the cultivation technology. Accordingly, a wide range of effects is noted on soil characteristics, crop growth and yield when SMS is used as a soil conditioner or fertilizer [104,105]. However, it is worth mentioning that the mushroom species and the SMS composition are often not specified in pertinent publications, making it difficult to draw sound conclusions about its exploitation prospects.

A summary of relevant reports on incorporating SMS into soils is presented in Table 3, which includes information on the SMS origin, type, and incorporation rate, and the main effects of SMS addition on the soil and plants under study. The presented results indicated improvements in soil structure and fertility, which led to increased crop production or contributed to the restoration of barren lands and degraded soils.

**Table 3.** Reuse of spent mushroom substrate (SMS) as soil amendment based on the outcome reported in pertinent publications. Abbreviations used: NPK, nitrogen, phosphorus, potassium NR, not reported; OC, organic carbon; OM, organic matter; PGPB: plant growth promoting bacteria; TN, total nitrogen.

Origin of SMS	Type of SMS and incorporation rate to soil in field or pot experiments	Effects noted on the soil and/or crops after the use of SMS	Reference
NR	SMS (10–50%) and poultry manure (10–50%) mixed with saline soil in pots	Increase of nutrient availability and salt-tolerant PGPB observed in treated saline soils; using 10% poultry manure and 10% SMS significantly enhanced maize plant growth and yield	[106]
*Agaricus bisporus*	SMS integrated to the soil in doses of 25 and 100 Mg ha^−1^ (dry weight)	SMS in degraded vineyard soils enhanced dehydrogenase activity, respiration activity and soil microbial biomass	[107]
*A. bisporus* and *Pleurotus ostreatus*	Composted *A.* *bisporus* SMS and *P.* *ostreatus* SMS 7:3 (v/v) to replace peat in pots	Higher yields of baby leaf lettuce, i.e. 3–7 times more than that obtained by peat (even under the pressure of the soil-borne plant pathogen *Pythium irregulare*)	[108]
NR	SMS (20 Mg ha^−1^) and chicken manure, applied to sandy soils every 1–2 years for 20 years	OM content increased; pH increased by 1–1.2 units, while soil bulk density decreased; the content of residual pores increased by 30–251%, and the fitted unsaturated hydraulic conductivity decreased	[109]
NR	SMS (35 g) in soil-containing pots (1.5 kg)	Increased NPK and OM contents, soil PGPB, and soil enzyme activities; higher biomass and chlorophyll content obtained in *Hibiscus sabdariffa* in comparison to the use of mineral NPK (16:16:16) fertilizer	[110]
*Flammulina velutipes*	Fresh or sterilized SMS (5%, w/w) mixed with soil in glass jars	Total and dissolved OC, microbial biomass carbon and nitrogen, abundance and diversity of bacteria and fungi, and enzyme activities were enhanced	[111]
*A. bisporus*	SMS (45 and 85 ton ha^−1^) mixed with soil in pots	SMS promoted the presence of fungi in the highly connected fraction of the active microbial community	[112]
*Auricularia auricula-judae*	Composted SMS, biogas residues and pig manure 1:1:1 in seedling pots	Better seedling quality was obtained by using the SMS-based substrate than with the commercial seedling substrates	[113]
*Volvariella volvacea*	Fresh, weathered, and carbonized SMS mixed with soil (1:2) combined with 0, 50 or 100% of the required rate of nitrogen fertilizer in pots	Weathered and carbonized SMS increased available N; fresh SMS immobilized various nutrients; high yields of pechay during first and second crop on weathered and carbonized SMS; fresh SMS led to high yields only during the third crop; yield was increased by N fertilizer only in weathered and carbonized SMS treatments	[114]
*A. bisporus*	Composted SMS (5 to 75 g L^−1^) in pots	SMS (as the sole fertilizer source) improved grass (*Lolium multiflorum*) yield up to 300% (with a concentration/dependent response) compared to the untreated control (with no NPK fertilization)	[115]
NR	SMS used to supply 50% or 100% of the crop’s nitrogen requirements	In contrast to mineral fertilizers, no increase in salt content was recorded when SMS was applied; similar lettuce and leek yields when either SMS or mineral fertilizers were used	[116]
*P. ostreatus*	Fresh SMS incorporated (15–20 t ha^−1^) during a period of four years to a depth of approx. 10 cm	SMS led to increase in porosity and fractal dimension, and caused strong development of a granular microstructure in the A horizon (15–20 cm) and a spongy structure in the B horizon (45–50 cm and 70–75 cm)	[117]
*A. bisporus*, and *A. bisporus* with *P. ostreatus* (1:1, v/v)	SMS was incorporated to a soil depth of 30 cm, 1 month prior to planting; both organic treatments providing 100 kg/ha of N	SMS amendment on a calcareous clayey-loam soil resulted in higher oxidizable OC, organic N, extractable K, and available P compared to soil fertilized by 100, 22 and 208 kg/ha N, P and K, respectively; the use of SMS provided lettuce yields similar to that obtained with mineral fertilizer	[105]
NR	SMS and peat moss alone or mixed (1:1, 1:2, and 2:1 (v/v)) with or without NPK fertilizer	SMS could replace up to 50% peat moss to support Chinese kale (*Brassica oleracea*) production; SMS alone cannot be used as growth medium because of its low nutrient content	[118]
*A. bisporus*, and *A. bisporus* with *P. ostreatus* (1:1, v/v)	SMS-based treatments provided 100 kg ha^−1^ of N	SMS increased the oxidizable OC, organic N, available P, respiration rate, and phosphatase activity, while it did not affect pH, EC, catalase, and urease activities in soil cultivated with lettuce	[119]
NR	Fresh or composted SMS applied annually for four years at rates of 8 and 25 Mg ha^−1^ (d.w.)	SMS led at increased OC, ΤΝ and labile organic forms as well as enhanced microbiological activity in a semiarid vineyard soil	[120]
*Agaricus subrufescens* and *Lentinula edodes*	*A.subrufescens* SMS (5 to 40%, d.w.) and *L.* *edodes* SMS (5 to 25%, d.w.) mixed with soil in pots	SMS led to increase of water retention and enhanced the soil microbial population; when supplemented by 10% of *A.* *subrufescens* SMS, lettuce dry weight increased by 2.2 and 1.3 times compared to the control and the NPK (44% N, 37% P2O5 and 48% K2O) treatments; fresh *L.* *edodes* SMS did not perform equally well	[104]
NR	SMS distributed onto field plots with a manure spreader at rates of 22.5, 45.0, and 90 kg m^−2^	Corn yields were significantly higher in SMS-amended plots, and the nitrogen content of both grain and stover was significantly higher than the control	[121]

By applying SMS of unknown origin (20 Mg ha^−1^) and chicken manure (10 Mg ha^−1^) in a sandy soil every one-two years for 20 years, Lipiec et al. [109] reported an increase in soil organic matter content by 102–201%. The experiment also resulted in a long-term increase in field water capacity caused by the augmentation of residual pores by up to 251%. Similarly, fresh or composted SMS applied annually for four years at two different rates (8 and 25 Mg ha^−1^) to a semiarid vineyard soil increased the content of inorganic N in the soil surface (0–5 cm) [120]. However, only the highest SMS addition rate improved soil organic carbon, total nitrogen, and labile organic forms at 0–5 and 5–15 cm soil depths.

In other large-scale applications, incorporating *A. bisporus* SMS into the soil (100 kg ha^−1^) increased oxidizable organic carbon, organic N, and available P content [119]. The values obtained for using *A. bisporus* SMS alone were higher than those resulting from incorporating a mixture of *A. bisporus* and *P. ostreatus* SMS (1:1, v/v). Both schemes of SMS addition resulted in increased phosphatase activity compared to unamended soil, while no alterations in the soil salinity or pH value were observed, and N mineralization was low. The same treatments also had positive effects when examined in a calcareous clayey-loam soil used for lettuce production [105]. In that study, application of SMS resulted in higher values of oxidizable organic carbon, organic N, extractable K, available P, and cation exchange capacity (especially when using *A. bisporus* SMS) than in soils receiving NPK fertilization, while lettuce yields were similar.

Ngan and Riddech (2021) reported the application of a mixture of SMS with plant growth-promoting bacteria (*Bacillus amyloliquefaciens*) in the cultivation of *Hibiscus sabdariffa* [110]. The study revealed an improvement in soil properties exceeding the effect exerted by NPK fertilization. Unfortunately, the lack of information on the SMS origin makes it difficult to compare the results with those of other relevant studies.

Testing fresh or sterilized *F. velutipes* SMS in cucumber cultivation resulted in a significant increase in total organic carbon, dissolved organic carbon, and microbial biomass carbon compared to NPK use and to no fertilization [111]. The study revealed higher levels of microbial diversity and enzyme activities for the fresh SMS-amended soil compared to soil treated with mineral fertilizer. Correspondingly, *A. bisporus* SMS amendment in soils increased bacteria and fungi co-occurrence, and the plant yield was positively affected by the relative abundance of microbial hubs [112]. Similarly, the application of *Agaricus subrufescens* and *L. edodes* SMS enhanced soil microbial population, and resulted in a remarkable increase in lettuce plants' dry weight compared with the results achieved with no fertilization or NPK treatments [104]. For several other crops, SMS application to the soil led to higher yields than those obtained by mineral fertilization [105,116,121].

Soil biological properties play a critical role in the maintenance of ecosystem functions, crops productivity enhancement, and at mitigating the adverse effects of pollutants. The beneficial effect on soil biological properties, including the structure of microbial communities and associated enzyme activities, is an attractive aspect of using SMS as an amendment. The main disadvantage of SMS is the state of stability/maturity which – if imperfect/immature – could hamper its wide agronomic use. However, this issue could be overcome by composting it, alone or mixed with other crop residues, under controlled conditions [115].

Several studies have revealed that using SMS as an ingredient in the composting process promotes the degradation of organic matter in mixtures with waste sludge, pig manure, corn stalks, and cow dung [122–124]. It has also resulted in the enhancement of the humification process [125], at reducing ammonia emissions [122,126], facilitating heavy metal passivation [125], and improving the quality of the final product [122,124]. Furthermore, using composted *A. bisporus* SMS alone as a substrate for the cultivation of *Lolium multiflorum* resulted in a yield improvement by up to 300% compared to the reference of NPK fertilization [115]. Co-composting of *Au. auricula-judae* SMS with biogas residues and pig manure led to the production of higher quality seedlings than those obtained from commercial substrates [113]. Substrates containing composted *A. bisporus* and *P. ostreatus* SMS resulted in increased yields of baby leaf lettuce, even in the presence of the soil-borne plant pathogen *Pythium irregulare* [108]. Adopting appropriate methodologies, such as the addition of enzymes or earthworms (vermicomposting), during the composting process should result in further improvement of the quality of composted SMS by promoting the beneficial effects of autochthonous bacteria, increasing ion-exchange capacity, decreasing total carbon and C/N ratio, and promoting the synthesis of nitrates [127–129].

In conclusion, the use of SMS as soil amendment has beneficial effects on soil fertility and structure. SMS presents a promising potential for substituting, at least partially, the use of mineral fertilizers in continuous crops and thus contributes at mitigating soil secondary salinization and acidification, and at avoiding nutrient imbalances and accumulation of toxic allelochemicals.

### 4.2. Use of spent mushroom substrate for plant-disease control

To deal with the negative repercussions of using chemical-based pesticides in agriculture, the application of environmentally friendly products for pest protection is crucial. Biocontrol agents, including live organisms and biological pesticides, are potential alternatives for controlling plant diseases. In contrast to chemical pesticides, biocontrol agents have little impact on non-targeted organisms; they do not leave behind any long-lasting harmful leachates and do not lead to the development of resistant microbial strains or insects. However, they often exhibit low-medium effectiveness and a shorter shelf life [130,131].

The bioactive compounds in SMS have antimicrobial properties [132], which could be exploited against plant pathogens. Although *in vitro* studies have shown the potential suitability of mushroom and mycelium extracts against plant pathogens [87,133,134], they do not necessarily reveal the *in vivo* effectiveness. SMS application has shown to be effective in suppressing plant disease incidence. Table 4 shows examples of reported research findings on using SMS for controlling plant pathogens and pests, and also includes the SMS origin, the plant – pathogen/pest system, and the main outcome of each study.

**Table 4.** Reuse of spent mushroom substrate for the control of plant pathogens and pests based on the outcome reported in pertinent publications. Abbreviations used: ACT, aerated compost tea; CT, compost tea; NCT, non-aerated compost tea; NR, not reported.

Origin of SMS	Plant – Pathogen/Pest (disease’s common name)	Main outcome	Reference
*Lentinula edodes*	Arabidopsis – *Alternaria brassicicola*	SMS chitin/cellulose nanofiber complex showed disease suppression and growth promotion	[135]
*Hypsizygus marmoreus, Pholiota microspora, Lyophyllum decastes*, *Auricularia polytricha*	Arabidopsis – *A.* *brassicicola* (cabbage’s leaf spot)	Antifungal volatile compounds emitted by the SMS suppressed fungal infection when incorporated into the soil (1:2, v/v)	[136]
*Pleurotus ostreatus*	Tomato – *Fusarium oxysporum* (fusarium wilt)	SMS bio-fortified with *Trichoderma asperellum* reduced disease severity by 21.2–84.3%	[137]
*P. ostreatus*, *Volvariella volvacea*	Pepper – *Ralstonia solanacearum*, *Phytophthora capsica* and *Meloidogyne* spp. (Ralstonia wilt, Phytophthora blight, root-knot nematode)	Biofertilizer (BF) mixed with composted SMS showed a significantly higher disease-control efficacy than BF alone (59 and 76% for *P. ostreatus* and *V. volvacea*, respectively, vs. 37% in plain BF)	[138]
*P. ostreatus, L. edodes*	Tomato – *Xanthomonas gardneri* (bacterial spot)	Polysaccharides extracted from SMS (1.5 mg mL^−1^) reduced bacterial spot severity by 50% on tomato cotyledons, leaflets, and five-leaf plants	[139]
NR	Eggplant – *F.* *oxysporum* and *R.* *solanacearum* (wilt of eggplant)	SMS, farmyard manure and earthworm compost (1:1:1, w/w) was the most effective combination at inhibiting disease incidence (66.9%)	[140]
*L. edodes*	Rice – *Pyricularia oryzae* (rice blast fungus)	Hot-water extract of SMS inhibited the germination of *Pyricularia oryzae* conidia	[141]
*L. edodes*	Pepper – *Phytophthora capsici* (Phytophthora blight)	SMS inhibited mycelial growth of *P. capsici*, suppressed the *Phytophthora* blight disease of pepper seedlings by 65% and promoted plant growth by more 30% compared to the control	[142]
*Lepista nuda*	Cucumber – *Pythium aphanidermatum* (cucumber’s damping off)	The combination of SMS with peat compost and peat moss reduced the incidence of *Pythium* damping-off up to 58% and promoted the growth of cucumber seedlings	[143]
*Hericium erinaceus*	Tomato – *R.* *solanacearum* (tomato wilt)	Water extracts of SMS suppressed tomato wilt disease caused by *R. solanacearum* by 85% in seedlings, and promoted growth of tomato plants	[144]
NR	Tomato – *P.* *capsici* (Phytophthora blight)	*In vitro* bioassays revealed that SMS-ACT reduced *P. capsici* growth by 50% while SMS-ACT with nutrients reduced it by 66.5%; in greenhouse trials, disease reduction was 6.4–73.4%	[145]
NR	Melon – *Didymella bryoniae* and Podosphaera *fusca* (gummy stem blight and powdery mildew)	SMS-ACT and NCT reduced the severity of *P. fusca*, while only a delay was observed in the growth of *D. bryoniae*	[146]
*L. decastes* *Pleurotus eryngii*	Cucumber – *Podosphaera xanthii*, *Cladosporium cucumerinum*, *Corynespora cassiicola* and *Pseudomonas syringae* (powdery mildew, cucurbits scab, Corynespora leaf spot and angular leaf spot)	Autoclaved water extract of SMS reduced symptoms caused by *P. xanthii* and *Ps. syringae* but not those caused by *C. cassiicola* and *Cl. cucumerinum*; a mixture of autoclaved SMS with soil (1:2, v/v), significantly reduced powdery mildew, scab and angular leaf spot diseases	[147]
NR	Bean – *F.* *solani*, *Rhizoctonia solani* and *Macrophomina phaseolina* (beans root rot)	Soil amendment with SMS-CT was highly effective in reducing root rot incidence caused by *F. solani*, *R. solani* and *M. phaseolina* at pre-emergence damping-off stage and after 45 days	[148]
*L. decastes*	Cucumber – *Colletotrichum lagenarium* (anthracnose)	A disease reduction (over 70%) observed in autoclaved and raw SMS incorporated into the soil (1:2, v/v with soil)	[149]
*Agaricus bisporus*	Tomato – *Septoria lycopersici* (leaf spot disease)	Plants grown on SMS-containing substrates were resistant to infections caused by *S. lycopersici*	[150]

Several studies on SMS-based biocontrol products against plant diseases concern *L. edodes*. The *in vitro* antimicrobial activity of *L. edodes* SMS [87,151], was further evidenced when hot water extracts were used to inhibit the germination of *Pyricularia oryzae* conidia in rice plants and to suppress the growth of *Phytophthora capsici* in pepper plants [141,142]. A chitin/cellulose nanofiber complex isolated from *L. edodes* SMS exhibited significant activity against *Alternaria brassicicola* in *Arabidopsis thaliana* plants [135]. *L. edodes* SMS-based biocontrol agents reduced the disease symptoms and promoted plant growth [135,142].

*P. ostreatus* SMS can provide another alternative to suppress plant diseases. Paddy straw-based *P. ostreatus* SMS, bio-fortified with *Trichoderma asperellum*, led to a remarkable reduction of the severity index of *Fusarium oxysporum*-induced disease, while it contributed to enhanced tomato growth [137]. The application of a polysaccharide extract from *P. ostreatus* SMS and discarded *L. edodes* mushrooms reduced by 50% the severity of bacterial spot caused by *Xanthomonas gardneri* in tomato cotyledons, leaflets, and five-leaf plants [139]. Phenolic-rich extracts from *P. ostreatus* SMS have been shown to prevent the development of the parasitic plant broomrape in faba bean cultivars [152], and to improve the rice growth and yield parameters [153]. In another study, mixing composted SMS from either *P. ostreatus* or *V. volvacea* with a biofertilizer exhibited higher control efficacy against *Ralstonia* wilt and *Phytophthora* blight diseases, than using the biofertilizer alone [138].

SMS from less widely cultivated mushrooms has also shown suppressive activity against plant diseases. Application of SMS from *Ly. decastes* and *P. eryngii* into soils used for cultivating cucumber resulted in protection against disease symptoms caused by *Colletotrichum orbiculare*, *Podosphaera xanthii*, *Cladosporium cucumerinum* and *Pseudomonas syringae* [147,149]. A protective effect against *Colletotrichum lagenarium* in cucumber plants was observed after spraying a water extract of *Ly. decastes* SMS [149]. The incorporation of *Ly. decastes* SMS into soil suppressed the lesions caused by *Al. brassicicola* in *Arabidopsis thaliana* leaves; this effect was attributed to SMS volatile components [136].

The results so far indicate that SMS richness in antimicrobial compounds in concomitance with its natural microbiome, including organisms suppressing soil-borne plant pathogens, are essential prerequisites for developing relevant plant-disease control products. However, further experimentation, including evaluation in large-scale greenhouse and field trials, is required to fully benefit from that potential toward a solid sustainable agriculture model.

### 4.3. Effects of SMS on nutritional value and secondary metabolites production in plants

Plant secondary metabolites, including vitamins, terpenoids and polyphenols, in fruits and vegetables are important for reducing risks of cardiovascular diseases and maintaining good health [154,155]. Those molecules exert a wide range of effects on the plant and associated organisms, and their production depends on various biotic and abiotic factors [156].

SMS application affects the content of secondary metabolites in plants. Vahid Afagh et al. [157] reported that the incorporation of *Agaricus* SMS leachates in sandy soil (up to 15% (v/v)) significantly increased the content of essential oil, proline, and soluble sugars in chamomile (*Matricaria chamomilla*) in comparison to plants grown on non-supplemented soil. Increasing the SMS leachate content enhanced K and Na absorption, whereas N and P uptake was not affected. Similarly, the addition of SMS leachate (20–60% (v/v)) in the soil led to increased content of essential oil components, chlorophyll, and antioxidant compounds in chamomile [158]. Application of SMS as an amendment in soils where basil (*Ocimum basilicum*) was cultivated, resulted in a two-fold increase in essential oil components, and in an enhancement of its content in micro- and macronutrients [159].

SMS use in the cultivation of vegetables demonstrated a wide range of effects on the various parameters, including product yield and quality. Applying a leachate of *P. ostreatus* SMS and *A. bisporus* SMS (10–25% (w/w)) to the soil increased the content of chlorophyll in pepper leaves, and that of carotenoids and protein in fruits [160]. Furthermore, *A. bisporus* SMS biofortified with *Trichoderma harzianum* inhibited lipid peroxidation and protein oxidation with a significant increase in total polyphenol and flavonoid contents in tomatoes, and enhanced Fe^2+/^Fe^3+^ chelating activity and superoxide anion radical scavenging activity compared to an SMS-free control [161]. Similarly, *P. ostreatus* SMS biofortified with *Trichoderma asperellum* improved morpho-biochemical and nutritional parameters, such as the content of chlorophyll, carotenoids, total soluble sugars, total soluble proteins, lycopene, β-carotene, and ascorbic acid, and antioxidant properties, of tomato plants [137]. Another study, using SMS from *A. bisporus* or *P. ostreatus* for replacing peat moss by 25–100% (w/w), reported that the effect of SMS on the macronutrient content of tomato, courgette, and pepper plants was species-dependent [162]. A proportional increase of N content with the increase of SMS ratio in the substrate was observed for pepper, whereas no significant effect was evident for courgette and tomato. In addition, increasing the incorporation volumes of SMS increased K content for courgette and pepper, but not for tomato. Last, courgette and pepper exhibited similar P content when grown on SMS-based substrates and a peat control, whereas P content in tomato seedlings grown on SMS-based substrates was lower than in plants grown on peat.

Although the scientific data on the effects of SMS on the nutritional value of edible and medicinal plants are still limited, the available results reveal SMS potential to increase the content of specific elements and secondary metabolites in plants.

## 5. Spent mushroom substrate as source of enzymes and bioactive compounds

Producing enzymes and different bioactive compounds is a reasonable way of SMS valorization. SMS-derived enzymes are of interest in industrial sectors, such as brewing, baking, starch-processing, leather, and textile industries, as well as in bioremediation and the emerging biofuel and biorefinery business. SMS-derived bioactive molecules have also the potential for application in the pharmaceutical, biomedical, feed, and food sectors.

### 5.1. Enzymes

SMS is a source of various enzymes that can be recovered by extraction with different solvent systems. Furthermore, SMS can be used as substrate for the cultivation of enzyme-producing microorganisms.

#### 5.1.1. Recovery of enzymes from spent mushroom substrate

**Figure 2.**
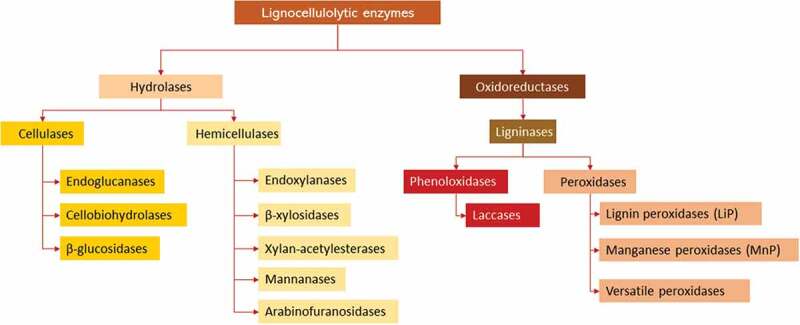
Enzymes participating in fungal degradation of lignocellulosic substrates.

For growing on lignocellulosic biomass, white-rot fungi secrete hydrolytic and oxidative enzymes responsible for degrading complex polymers into low-molecular weight substances, which can be assimilated for fungal growth [163]. The main groups of enzymes participating in fungal degradation of lignocellulosic materials are presented in Figure 2. Hydrolytic enzymes are responsible for deconstructing cellulose and hemicelluloses, while oxidative enzymes are involved in lignin degradation [7]. Consequently, upon the end of cultivation, SMS contains extracellular fungal enzymes, such as ligninases, cellulases, and hemicellulases, that can be recovered using different extraction procedures. The level of enzyme activities and their corresponding titers depend on the growth substrate and the fungal species’ ability to degrade different lignocellulose components. For example, since white-rot fungi degrade lignin and hemicelluloses preferentially, extracts of their spent substrates are rich in ligninases and xylanases, while cellulase activity is hardly detected.

The enzymatic systems present in SMS of various fungal species make possible their application for different purposes. For example, *P. ostreatus* SMS can be applied for decolorizing textile effluents because it contains oxidoreductases that degrade the dye molecules [164]. Similarly, the laccase and manganese peroxidase activities of *P. pulmonarius* SMS allow its direct application to remove polycyclic aromatic hydrocarbons from contaminated soil samples [165]. However, rather than directly using the bulk SMS, many applications require using isolated enzymes that can be recovered from SMS.

Table 5 shows an overview of studies on the recovery of extracellular enzymes from SMS of various fungal species. The spent substrates of *A. bisporus* and oyster mushrooms (*Pleurotus* spp.) are commonly reported as sources of extracellular enzymes. Xylanases and cellulases are the most common hydrolases in the recovered enzymes, while laccases are the main reported oxidoreductases. Most studies provide a relatively detailed description of the extraction process used, while purification protocols, e.g. dialysis, ultra-filtration, anion-exchange chromatography, or gel filtration, of the extracted enzymes are not always described in detail. Production of crude enzyme extracts and their application in areas where expensive purification can be avoided is often reported [164,181,182]. Some studies provide the exact identification of the extracted enzymes, including the complete EC classification number, while other provide trivial names or a more general classification without stating details.

**Table 5.** Recovery of enzymes from spent mushroom substrate. Abbrieviations used: PEG, polyethylene glycol; MnP, manganese peroxidase; LiP, lignin peroxidase; VP, versatile peroxidase.

Mushroom species	Hydrolases	Oxidoreductases	Extraction conditions	Comments	Reference
*Agaricus bisporus*	Endoxylanase, β-xylosidase, xylan-acetylesterase, arabinofuranosidase, endoglucanase, cellobiohydrolase, β-glucosidase	Peroxidase,phenoloxidase	Distilled water, 0.1 M NaOH, 0.1 M HCl, potassium phosphate buffer, different pH, 37°C, 1 h	No purification; the crude extract was effective for hydrolyzing wheat straw polysaccharides	[166]
*A.bisporus*		Laccase	Tris – HCl buffer, pH 7.5	No purification; the crude extract was used for oxidation of phenolic compounds	[167]
*A.bisporus*		Laccase	Distilled water, 4°C, 24 h	Purification by aqueous K_3_PO_4_-PEG two-phase system	[168]
*A.bisporus*	CMCase, xylanase, cellobiohydrolase	VP, MnP, LiP, laccase	Sodium citrate buffer, pH 4.8, room temperature, 150 rpm, 2 h	Acetone precipitation and DEAE chromatography; extract used for hydrolysis of SMS polysaccharides	[169]
*Pleurotus sajor-caju*	Xylanase, cellulase, β-glucosidase,	Laccase, LiP.	Tap water (pH 8.45), distilled water (pH 5.25), sodium citrate buffer (pH 4.0), 4 or 28°C, 200 rpm, 1–18 h	No purification	[170]
*Pleurotus ostreatus*, *Lentinula edodes*, *Hericium erinaceus*, *Flammulina velutipes*	α-Amylase, cellulase, xylanase, β-glucosidase	Laccase	1% NaCl, sodium phosphate buffer, tap water, room temperature, shaking, 1 h	No purification	[171]
*P.ostreatus*, *Pleurotus eryngii*, *Pleurotus cornucopiae*	α-Amylase, endoglucanase, endoxylanase	Laccase	Sodium citrate buffer, sodium phosphate buffer, tap water, distilled water, pH 4.5, 4–20°C, 200 rpm, 2–12 h	No purification	[172]
*P.eryngii*	Xylanase, β-xylosidase, β-glucosidase, α-amylase, cellulase	Laccase, LiP	Tap water, 1% NaOH, phosphate buffer, 4°C, 200 rpm, 2 h	No purification	[173]
*P.ostreatus*		MnP, laccase, LiP	Sodium tartrate buffer, pH 5.2, 22°C, 150 rpm, 2 h	Ultrafiltration; extract used for soil bioremediation	[174]
*Pleurotus florida*	CMCase, xylanase, cellobiohydrolase	VP, MnP, LiP, laccase	Sodium citrate buffer, pH 4.8, room temperature, 150 rpm, 2 h	Acetone precipitation, DEAE chromatography	[169]
*P.florida*	Xylanase	Laccase	Sodium citrate buffer, pH 4.8, room temperature, 2 h	Partial purification by ammonium sulfate precipitation and dialysis	[175]
*Pleurotus pulmonarius*		Laccase, MnP	Sodium citrate buffer, pH 4.8, 30 min	No purification	[163]
*P.pulmonarius*	Endoglucanase, xylanase, endoglucanase	LiP, laccase	Tap water, pH 4.0, 4°C, 150 rpm, 1 h	Concentration by freeze drying	[176]
*P.ostreatus*, *Pleurotus citrinopileatus*, *Auricularia auricula-judae*, *Coprinus comatus*, *Agrocybe cylindracea*, *He. erinaceus*, *Hypsizygus marmoreus*, *Tremella fuciformis*	Xylanase		Tap water, 25°C, 150 rpm, 3 h	No purification	[177]
*L.edodes*, *He. erinaceus*, *Stropharia rugosoannulata*, *Fomes fomentarius*, *Grifola frondosa*	Xylanase	Laccase	Water, 18–48 h, 10 or 20°C	Purification by ultrafiltration, stabilization of the 10 kDa retentate with either glycerol or maltodextrin/sodium benzoate	[178]
*L.edodes*, *P.ostreatus*, *P.eryngii*, *Pleurotus* spp., *F.* *velutipes*, *Hypsizygus marmoreus*	Total cellulase, CMCase, avicelase, β-glucosidase, dextranase, amylase	Laccase	Distilled water, 30°C, 180 rpm, 1 h	No purification	[179]
*Tremella fuciformis*	Xylanase, cellulase, pectinase		Tap water, 25°C, 150 rpm, 3 h	Ammonium sulfate precipitation, dialysis, DEAE chromatography	[177]
*Ganoderma lucidum*		Laccase	Sodium acetate buffer (pH 5.0), liquid-solid ratio 5, 4°C, 3 h	Partial purification by ammonium sulfate precipitation and dialysis	[180]

Recovery of extracellular enzymes from SMS was reported for the first time by Ball and Jackson in 1995 using *A. bisporus* spent compost [166]. In that study, it was found that lignocellulose-degrading enzymes can be recovered from spent mushroom compost by extraction with distilled water [166]. The evaluation of the enzyme activities revealed high levels of hemicellulases (endoxylanase, β-xylosidase, xylan-acetylesterase, and arabinofuranosidase), cellulose-degrading enzymes (endoglucanase, cellobiohydrolase, and β-glucosidase), and ligninolytic enzymes (peroxidase and phenoloxidase). The activity and stability of the enzymes suggested their potential for the biological upgrading of wheat straw. After Ball and Jackson’s pioneering study, *A. bisporus* SMS has been studied frequently to recover enzymes by extraction with different solvent systems [167–169]. Trejo-Hernandez et al. [167] reported laccase extraction with Tris – HCl buffer, while Mayolo-Deloisa et al. [168] developed a protocol using an aqueous potassium phosphate-polyethylene glycol two-phase system for purification of laccase extracted from *A. bisporus* SMS. Devi et al. [169] recently reported the recovery of oxidative and hydrolytic enzymes by suspending *A. bisporus* SMS in sodium citrate buffer, followed by acetone precipitation and subsequent chromatographic purification. The partially purified enzyme extract was evaluated on hydrolysis of SMS polysaccharides for ethanol production.

Recovery of enzymes from SMS resulting from the cultivation of mushrooms of the genus *Pleurotus* has been well investigated. The first studies were published in the early 2000s, when different solvent systems, including water, sodium citrate buffer, and sodium phosphate buffer, were evaluated to extract hydrolases and oxidoreductases from SMS of *P. sajor-caju* SMS [170] and *P. ostreatus* [171], the latter also included SMS of other species. In other studies, different buffers and conditions were evaluated for extraction of α-amylase, endoglucanase, laccase, and endoxylanase from SMS of *P. eryngii, P. ostreatus*, and *P. cornucopiae*; the best recoveries were achieved using sodium citrate buffer [172,173]. Sadiq et al. [174] used a sodium tartrate buffer for extracting manganese peroxidase (MnP), laccase, and lignin peroxidase (LiP) from *P. ostreatus* SMS and used the extract for bioremediation of contaminated soil. The SMS of *P. florida* [36,44] has also been reported as a source of lignin oxidases (versatile peroxidase (VP), MnP, LiP, and laccase) and polysaccharide hydrolases (CMCase, xylanase, and cellobiohydrolase). Crude extracts of *P. pulmonarius* SMS demonstrated laccase and MnP activity [163]. *P. pulmonarius* SMS was also used to extract several hydrolases and oxidoreductases, and the extract was used for the hydrolysis of palm oil mill effluent to produce biohydrogen [176]. Extraction of xylanases from *P. ostreatus* and *P. citrinopileatus* SMS has also been investigated [177].

The potential for enzyme recovery from SMS of other mushroom species has also been investigated. For example, Schimpf and Schultz (2016) screened selected enzyme activities in SMSs of *L. edodes*, *He. erinaceus*, *Stropharia rugosoannulata*, *Fomes fomentarius*, and *Gr. frondosa* and developed a protocol for recovery of lignocellulolytic enzymes from *L. edodes* SMS [178]. In another study, SMS from the cultivation of *Au. auricula-judae, Coprinus comatus*, *Agrocybe cylindracea*, *He. erinaceus*, and *H. marmoreus* have also been investigated as a source of xylanases [177]. Screening of enzymes extracted from SMS of *L. edodes*, *H. marmoreus, F. velutipes*, and three *Pleurotus* strains revealed higher activity of cellulose-degrading enzymes for *L. edodes* extract, while the extracts of *Pleurotus* strains displayed higher laccase activity and ability to decolorize Coomassie Brilliant Blue [179].

Recovery of laccase and several hydrolases from *L. edodes, P. ostreatus, He. erinaceus, F. velutipes* SMS has also been reported [171].

Enzyme preparations with high xylanase activity were obtained from extracts from *Tremella fuciformis* SMS purified by ammonium sulfate precipitation and gel filtration chromatography [177]. The purified enzyme showed good thermal stability and potential for saccharification of xylan contained in wheat bran, sugarcane bagasse, and other biomass residues. Optimal conditions for laccase extraction from *G. lucidum* SMS and utilization of the extract to remove toxic chemicals from an aqueous environment have also been reported [180].

#### 5.1.2. Using SMS as substrate for cultivation of enzyme-producing organisms

Since SMS is rich in nutrients and contains potential carbon sources, it can be used as a substrate for producing enzymes by cultivating enzyme-producing organisms. SMS has been used to cultivate fungi of the genus *Trichoderma*, the most relevant for industrial production of cellulases [183]. Production of cellulases requires a cellulosic substrate for inducing the enzyme system *Trichoderma* spp., which consists of endoglucanases, exoglucanases, and β-glucosidases [184]. Cellulose contained in lignocellulosic materials is a more suitable inducer than other alternatives, which are too expensive for industrial-scale use. Before cultivation of a cellulase producer, lignocellulose requires being pretreated, for example by a hydrothermal process [185], to remove lignin and facilitate enzyme access to cellulose. A drawback of hydrothermal pretreatment is that it leads to the formation of by-products, such as furan aldehydes, aliphatic acids, and phenolic compounds, which are inhibitors of microorganisms and enzymes [186]. Using SMS avoids the downsides of pretreatment since – during mushroom cultivation – lignin and part of the polysaccharides are degraded without forming inhibitors [187], and, therefore, the substrate is prepared for being used in microbial fermentations.

Enzyme production by microorganisms cultivated on SMS has been less investigated than the extraction of enzymes from SMS not subjected to a new cultivation cycle. *Pleurotus* spp. SMS are among the most common ones to produce enzymes by other organisms (Table 6). Some studies report using SMS as substrates for conventional enzyme producers, while in other studies, the enzymes are produced by edible mushrooms cultivated on SMS.

**Table 6.** Production of enzymes by fungal cultivation on spent mushroom substrate. Abbreviations used: SSF, solid-state fermentation; SmF, submerged fermentation.

Mushroom species	Enzyme-producing organism	Produced enzymes	Comments	Reference
*Auricularia polytricha*, *Pleurotus ostreatus*, *Auricularia nigricans*	*Trichoderma reesei*	Cellulase	Ultrasonic-assisted fermentation	[188]
*Agaricus bisporus*	*Trichoderma* spp., *Aspergillus niger*	Endoglucanase, endoxylanase, amylase, β-glucosidase	SSF, no nutritional supplements were used	[189]
*Pleurotus pulmonarius*	*Trichoderma asperellum*	1,4-β-cellobiohydrolase,carboxymethylcellulase, β-glucosidase	SSF	[163]
*Pleurotus florida*	*Trichoderma longibrachiatum*	Endoglucanase, exoglucanase, xylanase	SSF, SmF	[175]
*Pleurotus sajor-caju*	*Penicillium echinulatum*	Endoglucanase, β-glucosidase, xylanase	SmF	[190]
*P.florida*	*Aspergillus aculeatus*	Cellobiase	SSF, SmF	[175]
*P.ostreatus*	*P.ostreatus*, *P.pulmonarius*, *Ganoderma adspersum*, *Ganoderma resinaceum*, *Lentinula edodes*	Laccase	SSF of SMS supplemented with wheat bran and soybean flour	[191]
*P.ostreatus*	*P.ostreatus*, *P.pulmonarius*	Laccase	SSF of SMS enriched with wheat bran and soybean flour; the crude enzyme was used for dephenolization of wastewaters	[26]

*Trichoderma* spp. are among the most common conventional enzyme producers cultivated on SMS, but there are also some reports on *Aspergillus* and *Penicillium* spp. In a recent study, He et al. [188] reported the production of cellulases by *T. reesei* grown on corn cobs-based SMS from *Au. polytricha*, *Auricularia nigricans*, and *P. ostreatus*. In that study, cellulase production was more effective when using earlier ‘flushes’ of SMS than when several harvests were produced on the same substrate. The highest cellulase activity was obtained using the third flush of mushrooms of *Au. polytricha* SMS, particularly when the fermentation process was assisted with ultrasound. The study showed that higher cellulase activity could be obtained by cultivation on untreated SMS than on acid- or alkali-treated SMS. The potential of spent mushroom compost (SMC) of *A. bisporus* for cultivation of enzyme-producing fungi has also been shown. Production of endoglucanase, endoxylanase, and β-glucosidase using *Trichoderma* isolates and a strain of *Aspergillus niger* on *A. bisporus* SMC without nutrient supplementation was reported [189]. SMS resulting from growing *P. sajor-caju* on sugarcane bagasse was used to produce cellulases and xylanases by *Penicillium echinulatum* [190].

The production of enzymes by cultivating edible mushrooms on SMS has also been reported. *P. ostreatus* SMS supplemented with wheat bran and soybean flour was a suitable substrate for the cultivation of *P. ostreatus*, *P. pulmonarius*, *Ganoderma adspersum*, *Ganoderma resinaceum*, and *L. edodes* for producing laccase [191]. The study showed good potential of the supplemented SMS for laccase production by *Ganoderma* spp. and fruitbodies by *Pleurotus* spp. In another study by the same group, laccase was produced by cultivating *P. ostreatus* and *P. pulmonarius* on *P. ostreatus* SMS, and the crude enzyme’s potential for removing phenolic compounds from olive mill and winery wastewaters was evaluated [26].

Another approach is cultivating enzyme producers in SMS that has already been subjected to the extraction of extracellular enzymes. This approach has been tested for *P. pulmonarius* SMS, which was first subjected to extraction of lignin-degrading enzymes, and then used as substrate for producing cellulases by *Trichoderma asperellum* cultivation [163]. It was also applied for *P. florida* SMS, which was first used as a source of laccase and several hydrolases, and then directed to the production of cellulases by either *Trichoderma longibrachiatum* and *Aspergillus aculeatus* [175]. *T. longibrachiatum* resulted in higher activity of endoglucanase, exoglucanase, and xylanase, while *As. aculeatus* was a better cellobiase producer (Table 6).

### 5.2. Bioactive compounds

SMS contains bioactive compounds of different functionality and origin. The fungal mycelium contains polysaccharides, sterols, proteins, polyphenols, vitamins, and other bioactive molecules. Mycelial growth throughout the surrounding environment also results in the secretion of potentially useful bioactive compounds. In addition, the extractive fraction of the lignocellulosic substrate and the oligomeric products from fungal degradation of polysaccharides and lignin might also be sources of bioactive substances. However, while the bioactive molecules of the sporocarps of edible fungi have been extensively investigated [192], the information on the bioactive potential available in SMS is still limited. Recovery of bioactive compounds is a promising direction for valorizing SMS.

#### 5.2.1. Polysaccharides

Polysaccharides are among the bioactive substances responsible for the immunomodulatory and antitumor effects of edible and medicinal mushrooms [193]. However, in most of the research dealing with fungal polysaccharides the investigated sources are fruitbodies or mycelia [194], while extraction from SMS has been less explored. A study on extraction and characterization of a polysaccharide from *L. edodes* SMS published in 2012 by Zhu et al. provided the start for the research on SMS as a source of bioactive molecules [195]. Henceforth, several relevant reports on obtaining bioactive extracts from SMS have been published. Most publications deal with *L. edodes*, *Pleurotus* spp., and *Ganoderma* spp., but SMS from the cultivation of other fungal species has also been investigated (Table 7).

**Table 7.** Recovery of bioactive compounds from spent mushroom substrate. Abbreviations used: UAE, ultrasound-assisted extraction; SSF, solid-state fermentation.

Mushroom species	Chemical	Production method	Comment	Reference
*Lentinula edodes*	Polysaccharide composed of anhydrorhamnose, anhydroglucose, and anhydromannose	Water extraction (90°C, 1 h), alcohol precipitation, centrifugation, freeze-drying, Sevag deproteinization and gel-filtration chromatography	Heteropolysaccharide with antibacterial activity	[195]
*L.edodes*	Partially hydrolyzed polysaccharides	Water extraction (85°C, 3 h), alcohol precipitation, centrifugation, hydrolysis with 1 M H_2_SO_4_, anion-exchange and gel-filtration chromatography	Antioxidant, anti-inflammatory, and renoprotective effects	[57,196]
*Ganoderma lucidum*	Polysaccharides	Water extraction, Sevag deproteinization and gel-filtration chromatography	Antioxidant activity	[197]
*Panus strigellus* (syn. *Pleurotus tubarius*), *Pycnoporus sanguineus*	β-Glucans, steroids, saturated triterpenes	Extraction with deionized water at 100°C	The extracts were concentrated by lyophilization before analyses	[198]
*Pleurotus eryngii*	Polysaccharide-protein complex containing 99% carbohydrate and 1% protein	Extraction with 0.5 M NaOH (90°C, 300 min), ethanol precipitation, dialysis, Sevag deproteinization, dialysis, and gel-filtration chromatography	Strong antioxidant activity	[199]
*Cordyceps militaris*	Polysaccharides	Partial enzymatic hydrolysis (45°C, pH 4.0, 2 h), gradient ethanol precipitation, Sevag deproteinization	Antioxidant activity with no cytotoxicity	[200]
*Agrocybe cylindracea*, *L.edodes*, *Hypsizygus marmoreus*, *Pleurotus ostreatus*, *C.militaris*	Polysaccharides	Enzymatic extraction, ethanol precipitation, Sevag deproteinization	Antioxidant activity	[201]
*P.ostreatus*	Crude exopolysaccharides	SSF of *P.* *ostreatus* and *P.* *pulmonarius*, EPS extracted with water at 60°C, 15 min	SSF produced laccase, fruitbodies and crude EPS	[191]
*L.edodes*	Acid polysaccharides	Extraction with .5 M KOH (90°C, 300 min), ethanol precipitation, Sevag deproteinization, purification by gel-filtration chromatography	Heteropolysaccharides with antiproliferative activity against human tumor cells	[193]
*P.ostreatus*	Crude polysaccharides	Autoclaving with distilled water (120°C, 30 min), ethanol precipitation	Protective effect against plant diseases	[139]
*L.edodes*	Ergosterol, ergosta-7,22-dienol, β-sitosterol	UAE, optimal conditions: 432 W, 16 min, liquid-solid ratio 22	Antitumor activity	[58]
*H.marmoreus*	Pentostatin	SSF by *C.* *militaris*	Pentostatin is a powerful anticancer drug	[202]

UAE, ultrasound-assisted extraction; SSF, solid-state fermentation.

Water extraction at temperatures around 80–90°C, followed by alcohol precipitation, and chromatographic purification, is a standard procedure for recovering polysaccharides from SMS. Accordingly, a heteropolysaccharide displaying antibacterial activity against three different microorganisms was recovered from *L. edodes* SMS [195]. The same method, combined with partial hydrolysis, either chemical [57] or enzymatic [196], was applied to *L. edodes* SMS for extracting polysaccharides showing antioxidant, anti-inflammatory, and renoprotective activities. Water extraction has also been reported to extract polysaccharides from *G. lucidum* SMS [197], and to extract β-glucans and other compounds from rice husk-based SMS of *Pycnoporus sanguineus* and *Panus strigellus* (syn. *Pleurotus tubarius*) [198].

Extraction with aqueous alkaline solutions is another useful method for recovering polysaccharides. He et al. [199] reported the obtention of a polysaccharide extract from *P. eryngii* SMS by alkaline extraction followed by deproteinization and gel filtration chromatography. The refined product was a polysaccharide-protein complex containing 99% (w/w) of a polysaccharide composed of anhydroxylose, anhydroglucose, and anhydroarabinose units. Strong antioxidant activity – with potential food applications – was revealed *in vitro* for the polysaccharide-protein complex. A comparable extraction approach has also been used to recover polysaccharides from *L. edodes* SMS [193]. Exhaustive characterization revealed that the *L. edodes* SMS extract contained heteropolysaccharides exerting antiproliferative effects against six tested human tumor cell lines.

Subcritical water extraction (SWE) can be applied to extract bioactive molecules. SWE of polysaccharides from *P. ostreatus* SMS and *L. edodes* residual basidiocarps by autoclaving at 120°C has been reported [139].

Partial enzymatic hydrolysis can also be used to extract polysaccharides from SMS. Hydrolysis with cellulases for two hours was used for recovering polysaccharides from the SMS of *C. militaris* [200]. Four polysaccharide fractions were isolated, and three displayed good antioxidant activity with no cytotoxicity. Enzyme treatment has also been used to recover polysaccharides from SMS of *Ag. cylindracea*, *L. edodes*, *H. marmoreus*, *P. ostreatus* and *C. militaris* [201]. The polysaccharides were isolated from the extracts by ethanol precipitation and purified by deproteinization with the Sevag regent, and their antioxidant activity was evaluated *in vitro*. The polysaccharides from *Ag. cylindracea* SMS had the best oxygen free radical-scavenging capacity and ferric reducing power (FRAP), while those from *Hy. marmoreus* and *P. ostreatus* displayed the best ABTS and DPPH radical scavenging activities.

Another way of producing chemical compounds of interest is to use SMS as a substrate for cultivating other organisms. For instance, *P. ostreatus* SMS was reported to be used for producing crude exopolysaccharides by cultivation of *P. ostreatus* and *P. pulmonarius* [191].

#### 5.2.2. Sterols and other compounds

Ergosterol, the most abundant sterol in fungi, has relevant biological activities for food, pharmaceutical, and biomedical uses, and it is a precursor of vitamin D_2_. Most of the reports on ergosterol extraction from mushroom residues deal mainly with stipes of fruitbodies or mushrooms not meeting commercial specifications [203]. However, the potential of *L. edodes* SMS as a source of ergosterol has recently been demonstrated [58]. Ergosterol-rich extracts were obtained from *L. edodes* SMS using ultrasound-assisted extraction, a non-conventional technique for extracting natural products from various biomaterials; *in vitro* experiments revealed that the produced extracts have antitumor activity against three cancer cell lines.

The presence of steroids and saturated terpenes has been shown in water extracts of SMS from the cultivation of *Py. sanguineus* and *Pa. strigellus* (syn. *P. tubarius*) SMS on rice husk [198]. The purine analog pentostatin, a potent anticancer drug, was produced by cultivating a cellulose-degrading transformant of *C. militaris* using *H. marmoreus* SMS as substrate [202].

Phenolic compounds can also be extracted from SMS. Elsakhawy et al. reported the production of phenol-rich extracts from *P. ostreatus* SMS using either 0.5 N NaOH [152] or tap water [153] as solvents. The produced extracts were further assayed as plant-disease control and biofertilizer.

## 6. Spent mushroom substrate valorization as part of cascade use of plant biomass

**Figure 3.**
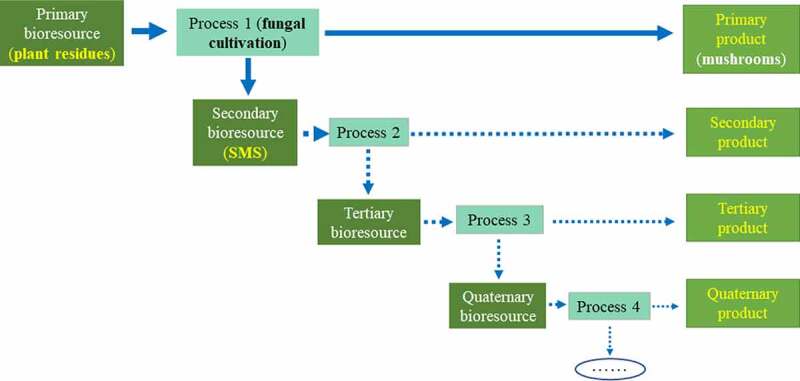
Schematic illustration of the cascade-use concept of bioresources (based on Vis et al. 2016 [204], p. 6, https://data.europa.eu/doi/10.2873/827106).

The generation of plant biomass resources by agriculture and forestry takes a long time and requires considerable land areas; thus, their utilization should be rational and efficient. Residual biomass materials, such as side/waste streams or byproducts from varying stages of production/processing chains, contain components of high potential for value-added applications. A common approach for biomass valorization today is to burn it in a resource-inefficient way to generate heat and power for energy purposes. For bioeconomy development in a resource-efficient way, cascade use of plant biomass should always be considered. Cascade use, also known as cascading use [204], is a complex interaction of material flows used as a strategy to increase resource efficiency in biomass processing. Cascade use occurs when biomass is processed through a series of material uses (Figure 3), by reuse and recycling, before finally being used for energy recovery [205].

Cultivation of edible mushrooms plays a unique role in supplying highly nutritive and health-promoting food. Still, it generates vast amounts of SMS, which is mostly discarded or inefficiently used despite its potential for generating value-added products. As a cellulose-rich bioresource, SMS can also be considered a material of interest for developing sugar-platform applications after enzymatic saccharification. Compared with the lignocellulosic materials used to formulate the initial mushroom substrate, SMS is more susceptible to biochemical conversion using enzymes and microorganisms [16,187,206]. That is mainly because the cultivation of white-rot edible fungi constitutes a biological process that modifies lignocellulose by removing a large part of lignin and hemicelluloses, which interferes with the enzymatic saccharification of cellulose. Furthermore, SMS, i.e. a material resulting from the aforementioned process (which could be considered as a biological pretreatment), contains few inhibitory compounds or external chemicals that might negatively affect downstream processing or harm the environment [187,206].

Applying cascade uses to processing plant biomass by mushroom cultivation combined with SMS valorization through biochemical conversion and other approaches, is expected to maximize the cost-effectiveness of a value chain of variable potential products. The cascade-use concept also results in minimizing resource loss and environmental impacts. Following a cascading approach, SMS, as the primary by-product of mushroom cultivation can be re-used as raw material for new processes, extending total biomass availability within the system. That is a rational approach, where different valuable biomass constituents are recovered and converted into value-added products. Energy uses of residual biomass are considered only at the end of the life cycle when all higher-value products and services have been exhausted. There are different possible examples of multi-stage cascading uses for SMS valorization. Three promising published case studies are discussed in this section.

### 6.1. Case study 1: food – ethanol – solid fuel

**Figure 4.**
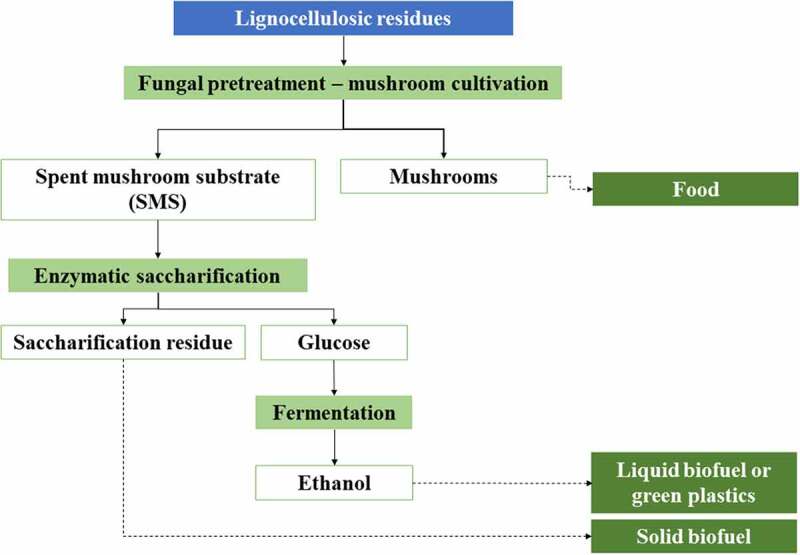
Schematic illustration of the concept food – ethanol – solid fuel. The figure is modified from Chen, 2021 [208], p. 15, https://pub.epsilon.slu.se/26324/1/chen_f_211216.pdf.

The integrated production of *L. edodes* mushroom (food) and biofuels from hardwood residues can be an example of cascade use [16,187,206,207]. Food (mushrooms) is produced on a lignocellulosic substrate. Concomitantly, mushroom cultivation selectively degrades lignin and hemicelluloses, thus facilitating the enzymatic saccharification of cellulose. Glucose from the saccharification process can then be fermented to ethanol using yeast. Enzymatic saccharification also generates lignin-rich solid leftovers, which can be used as a solid fuel (Figure 4).

From a circular bioeconomy point of view, the forest residues can be considered primary bioresources from forest production (c.f. Figure 3). Exploiting the forest residues as mushroom growing substrates results in the production of fruitbodies as primary products. SMS is the secondary bioresource and can be converted to the secondary product ethanol. Ethanol can be used to synthesize renewable polyethylene to produce green plastics or for fuel applications, including advanced jet biofuels. After cellulose saccharification, the solid leftover can also be recovered as a tertiary bioresource/biowaste, and converted to solid fuel, a ‘tertiary product.’ A recently published mass balance analysis revealed that one ton of birch-based initial mushroom substrate might result in about 600 kg of fresh shiitake (*L. edodes*) fruitbodies (90% moisture), 130 liter of ethanol, and 300 kg (dry mass) of solid biofuel [187]. This system/approach can also be applied to other mushroom species. Using an experimental setting like the one used for *L. edodes*, Chen et al. [209] found that one ton of birch-based initial substrate might result in about 400 kg of fresh fruitbodies (90% moisture) of *Au. auricula-judae*, 35 liters of ethanol, and 300 kg dry mass of solid biofuel. The solid fuel was found to have a relatively high calorific value and favorable characteristics for direct combustion to produce heat. The generated heat can be used for the pasteurization of substrates or space heating.

The outcome of the production chain can be affected by the composition of the initial substrates used for mushroom cultivation. Chen et al. [206] reported that alder-based substrate led to 4% more mushroom fruitbodies, 14% more ethanol, and 23% more solid fuel than birch-based substrate. On the other hand, an aspen-based substrate resulted in a 37% lower yield of fruitbodies than the birch-based one, although the yields of ethanol and solid fuel were comparable for substrates from both tree species.

The same concept is also applicable to other biofuels. For instance, another ‘food – biofuel – solid fuel’ alternative is to produce biogas instead of ethanol as a secondary product. Lin et al. [210] cultivated shiitake on woodchips and produced biogas by anaerobic digestion (AD) of SMS; at the end, around 53–57% (dry mass) of the substrate was solid leftover. Since the AD process consumes mostly carbohydrates [211,212], the leftovers are expected to have a relatively high content of lignin, a component with a high calorific value. Therefore, using the leftovers as a solid fuel for a self-supporting heating system could be meaningful. In a slightly different alternative, rice straw was used as the main ingredient for *P. ostreatus* substrate, fruitbodies were produced as the primary product, SMS was directed to AD for producing biomethane as a secondary product, and the AD digestate was used as biofertilizer (tertiary product) for rice cultivation [213].

### 6.2. Case study 2: food – biogas − 2^nd^ cycle mushroom

Another example of a cascade system was reported by Ikeda et al. [214] using the mushroom ‘enokitake’ (*F. velutipes*) cultivated in a substrate based on corncobs supplemented with rice bran. In this case, agricultural residues were the primary bioresource, and enokitake fruitbodies were the primary product. The SMS resulting after mushroom harvest was anaerobically digested for producing biogas, the secondary product. After the AD process, around 45% of the initial mass was left as solid residue or digestate. In the next step, KOH or NaOH was used for pretreating the AD residue (SMS-ADR), which was then mixed with rice bran at a 50:50 weight ratio for formulating a new substrate to be subsequently used for a second enokitake cultivation cycle. The results were promising: the yield of the tertiary product, i.e. mushrooms cultivated on SMS-ADR, was comparable to those of the primary one, i.e. mushrooms cultivated on corncob-based ‘standard’ substrate. Crude protein, ether-extracted compounds, crude fiber, minerals (Na, P, Ca, K, Mg), and free amino acids in fruitbodies showed similar content to those obtained from the standard substrates. The study did not discuss further use of the second cycle SMS (SMS-II). In our opinion, this cascading system could still be extended to a quaternary product by exploiting the potential of SMS-II as a biofertilizer or a soil amendment.

Cascade systems including biogas as the secondary product are also feasible for other mushroom species. Since lignin content decreases during the mushroom cultivation, the resulting SMS is accessible to anaerobic microbes, thus facilitating AD conversion of carbohydrates to biogas. On the other hand, after biogas production, and although data on the chemical composition of the digestate are not available [215], the lignin content is expected to increase due to its recalcitrance to degradation by anaerobic bacteria [211,212,216]. Therefore, it was wise to choose white-rot fungi again to break the recalcitrance of lignin to produce additional value-added tertiary products. Although the low pH and the presence of unknown by-products may inhibit a second mushroom cultivation cycle, it was shown that KOH or NaOH soaking was a viable method to improve the susceptibility of digestate to be further used for enokitake cultivation [214]. Nevertheless, the precise mechanism behind the alkaline reactivation of the digestate from AD for mushroom cultivation remains to be clarified.

### 6.3. Case study 3: food − 2^nd^ cycle mushroom – enzymes

Another possible cascading chain can include two cycles of mushroom cultivation in a row followed by recovery of extracellular enzymes as a tertiary product. Economou et al. [191] reported a case study, where oyster mushroom (*P. ostreatus*) was produced on a wheat straw-based substrate, and the resulting SMS (SMS-I) was tested as the main ingredient of the substrate for a second mushroom cultivation cycle. After harvesting the fruitbodies from the second production cycle, the generated SMS (SMS-II) was used as a source for the recovery of the lignin-degrading enzyme laccase. Among the five fungal species tested for the second mushroom production cycle, *P. pulmonarius* resulted in the SMS providing the highest yield of laccase, 2465 U g^−1^ day^−1^ (dry mass based). The crude laccase extract was then used for the dephenolization of wastewaters [26]. The authors did not explore possible uses of the solid stream remaining after laccase extraction from SMS-II. A potential extension of the cascading system would be possible by using that stream as either biofertilizer or solid fuel.

Using *P. ostreatus* as the species involved in the first step of the cascading system is a reasonable strategy considering that *Pleurotus* spp. are among the most studied white-rot fungi for biological treatments of lignocellulosic materials [217]. Compared with other edible fungi, they have the advantages of presenting a relatively shorter life cycle, and broader adaptation to substrate assortments and growing environments. Even though *Pleurotus* lignocellulolytic enzyme activities are generally comparable to those of *L. edodes* [215], the lignocellulose degradation capacity of the former is generally lower than that of shiitake, probably because of the shorter life cycle [217]. It must be emphasized that the determination of SMS composition, which is essential for fully understanding the potential of the SMS to be directed to new cultivation cycles, is often underestimated in the literature.

The cultivation of white-rot edible fungi on primary bioresources results to food (mushrooms), and functions as a biological pretreatment for facilitating biochemical conversions. Therefore, mushroom cultivation is crucial in cascading systems of lignocellulosic biomass utilization. In addition to the case studies discussed above, many other cascade systems producing fruitbodies as a primary product, and including other products or services, can be proposed to valorize SMS and other residual streams. The feasibility of producing antibiotics [218], antitumor sterols [58], seedbed of vegetables [219], fertilizers [104], soil bioremediation agents [220], enzymes [175], biochar [221], and other products has been demonstrated. Some products could be considered as different ‘puzzle pieces’ to be chosen and integrated into a chain of cascade uses. However, appropriate approaches ensuring optimal process integration remain to be developed. Process integration has to be developed through interdisciplinary approaches to maximize system values for the circular bioeconomy and the protection of the environment. Furthermore, systematic evaluation (e.g. life cycle assessment) and optimization of processes for the cascading uses must be addressed.

## 7. Future directions and conclusions

Cultivation of edible and medicinal mushrooms is a very dynamic business, with an impressing development during the last decades. However, increased mushroom production leads to the generation of high quantities of spent mushroom substrate (SMS). The accumulation of non-used SMS, or its limited or not high-added value applications, undermines the future of pertinent commercial activities. Therefore, achieving an efficient valorization of SMS – beyond its current low-value use – is of paramount importance for the sustainable development of the mushroom industry. The research discussed in this review shows the vast potential of SMS as a source of valuable products and services.

The presence of valuable nutrients and energy sources for supporting new cultivation processes make SMS a suitable substrate component for new mushroom cultivation cycles provided that a suitable treatment or supplementation is applied. Reusing SMS in new cultivation of mushrooms of either the same or other species has significant potential for reaching high yields in an environmentally sustainable way and at the same time contribute to the reduction of production costs. The high nutritional value of SMS could also be exploited for the development of new feeds; the output of recent experimental work convincingly shows the feasibility of including SMS in the diets of poultry, ruminants, and monogastric animals, as well as beyond traditional husbandry, in pisciculture and insect farming. However, making SMS a regular diet ingredient poses complex challenges related to its fiber content and digestibility, and the acceptance by the animal. Recent research has faced those downsides since it has been demonstrated that by application of appropriate treatments the nutritional and acceptance features of SMS can be enhanced.

SMS’s physical properties and chemical composition support the development of novel environment-friendly and cost-effective bio-based products, which can be used as part of sustainable agronomic practices in substituting fossil-based fertilizers and synthetic pesticides. Well-designed experiments have shown that SMS application as a soil amendment or fertilizer has beneficial effects on soil fertility and structure, without causing secondary salinization or acidification. The reported research also shows the potential of SMS as source of products for biological control against plant diseases, and its favorable effect on the production of secondary metabolites in plants and at enhancing the nutritional value of the fruits and vegetables. Scaling up the experimentation to large-scale greenhouse and field trials is required. Furthermore, increasing demonstration actions are expected to fully demonstrate/evidence the potential of SMS within a sustainable agriculture model.

SMS contains extracellular enzymes secreted during fungal growth and used to degrade the substrate’s macromolecules. Those enzymes make it possible to use the SMS in services such as decolorization of textile effluents, bioremediation of contaminated soil, and wastewater treatment. Enzymes can also be extracted from SMS using different solvent systems. Furthermore, the potential of SMS as substrate for the cultivation of enzyme-producing microorganisms has been shown. The crude extracts of SMS enzymes can be subjected to various degrees of purification rendering refined preparations suitable for added-value applications, where enzyme purity is a decisive criterion.

Several publications report on exploiting SMS bioactive compounds for various uses. However, the extraction of bioactive compounds from SMS is just an emerging area of high interest. Recently published results indicated that SMS bioactive molecules could be used as added-value, sustainable, bio-based ingredients in socially-sensitive business sectors. SMS-derived nutraceuticals, food supplements, functional foods, and active ingredients might be the foundation of a new ‘next-generation mycotherapeuticals’ sector. That would require developing appropriate protocols for extracting bioactive molecules from SMS, a task that faces major challenges regarding the extraction’s effectiveness without affecting the properties of the molecules of interest, and by avoiding the degradation of non-targeted compounds that might also be of interest.

Applying the cascade-use concept to SMS valorization is essential to increase resource efficiency in biomass processing and mushroom production. Arranging different alternatives of SMS utilization in cascading systems, where mushroom production is included as the primary process and the by-products are converted to value-added products, will result in a value chain with minimal resource losses and with no adverse environmental impact, in agreement with the principles of sustainable development. Appropriate implementation of the cascade-use concept requires significant efforts to ensure optimal process integration based on interdisciplinary approaches. By achieving it, the system values can be maximized, and the extensive use of SMS for generating high-value products and services within a circular bioeconomy scenario can become a reality.

## Data Availability

Since no new data were created by the authors, data sharing is not applicable regarding their own research results. Raw data on search criteria used for building the review, as well as the excluded material, will be available upon reasonable request.
